# Single‐cell Transcriptome Profiling Reveals Gene Regulatory Networks and Key Genes in the Root Epidermis and Cortical Cells Associated with Early Nodulation in *Glycine Max*

**DOI:** 10.1002/advs.76550

**Published:** 2026-07-11

**Authors:** Yongbin Zhuang, Yu Geng, Xinyu Guo, Chen Wang, Yongjing Sun, Luqi Liu, Li Liu, QianQian Gao, XinFang Zhu, Hongxiang Cao, Gengshuo Zhang, Yunxia Liu, Guobin Zhang, Baoyin Chen, Jinfei Zhang, Xin Hou, Xiaoming Li, Dajian Zhang

**Affiliations:** ^1^ College of Agronomy Shandong Agricultural University Tai'an Shandong China; ^2^ Northeast Institute of Geography and Agroecology Chinese Academy of Sciences Changchun Jilin Province China; ^3^ The Key Laboratory of Plant Development and Environmental Adaptation Biology Ministry of Education College of Life Sciences Shandong University Qingdao Shandong China; ^4^ College of Life Sciences Shandong Agricultural University Tai'an Shandong Province China; ^5^ College of Plant Protection Shandong Agricultural University Tai'an Shandong Province China

**Keywords:** Glycine max, Nod19, nodule organogenesis, root nodule, ScRNA‐seq, WRKY

## Abstract

The legume crop soybean forms a symbiosis with rhizobia to fix atmospheric nitrogen (N) in specialized organs called root nodules. However, the mechanisms regulating early infection of the root epidermis and nodule‐primordium formation in the cortex for proper nodule formation remain unclear in soybean. Here, we report a single‐cell transcriptome analysis of mock‐ and rhizobia‐inoculated soybean roots at 4 days after inoculation, an important control point for autoregulation of nodulation and nodule‐primordium formation. We profiled 21,500 cells and detected 12 major cell clusters, and identified 193 infected‐cell‐specific, 205 epidermis‐specific and 180 cortex‐specific DEGs. Gene‐ontology enrichment and gene‐regulatory network analyses uncovered key pathways such as reactive oxygen species‐mediated hormone signaling involved in coordinating defense signaling and symbiotic pathways. We also identified and functionally validated an ethylene‐activated circuit comprising *GmWRKY6.3/6.4* transcription factors and select downstream *GmNod19* targets, in which genes act as positive regulators by promoting infection‐thread formation during early nodulation, thereby shaping nodule formation. This study showcases how single‐cell transcriptomics and gene‐regulatory networks provide hypotheses for identification and characterization of previously unappreciated regulatory circuits, broadens our understanding of precise genetic control underlying symbiosis establishment, and underscores how functional diversification of nodulation genes has occurred across legumes.

## Graphical abstract

Single‐cell transcriptomics of soybean roots soon after rhizobial inoculation reveals epidermal and cortical cell‐specific programs and gene‐regulatory networks acting in symbiosis establishment. We identify an ethylene‐driven regulatory circuit involving WRKY6.3/6.4 transcription factors targeting select *Nod19* genes that promotes infection‐thread formation, uncover regulators of legume–rhizobium symbiosis and provide data for hypothesis generation.

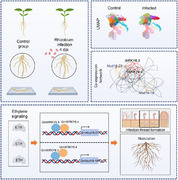

## 1 Introduction

Soybean (*Glycine max* L. Merrill) is the most economically important legume worldwide, providing more than 50% of the total edible oils consumed globally, and is also the principal source of vegetable protein worldwide for food and feed [1]. However, current productivity is not sufficient to meet the increasing demand for soybeans and contemporary production systems favor mitigating reliance on synthetic nitrogenous fertilizers to lift crop yields.

The legume–rhizobium symbiosis evolved approximately 58 million years ago [2, 3], leading to the organogenesis of specialized root nodules accommodating rhizobia that reduce inorganic N from the atmosphere into forms of organic N useable by the host plant [2]. Tremendous progress has been made in identifying genes underlying symbiotic nitrogen fixation (SNF), especially those involved in early signaling steps, mainly through screening for mutants with no or excess nodules in barrel clover (*Medicago truncatula*, 2*n* = 16) and *Lotus japonicus* (2*n* = 12). More than 200 genes have been reported to control host–rhizobium symbiosis across legumes, and approximately half of these were identified in *M. truncatula*, the preferred model species for indeterminate nodule development. By comparison, fewer genes have been identified in soybean, which forms determinate nodules; most of these genes are thought to participate in nodule organogenesis based on their functional annotation, leaving the regulatory basis of early infection events in determinate nodulation less well defined [4].

Although the functions of orthologous genes are generally considered to be conserved among legumes, species‐specific regulation of certain processes is also crucial for successful establishment of SNF [5, 6]. For example, *Isoflavone synthase* (*GmIFS*) is required for nodule formation in soybean, while knocking‐down its counterpart *MtIFS* has little effect on nodulation in *M. truncatula* [7, 8]. Certain other nodulin genes, such as *Interacting protein of does not make infections 3* [*DMI3*]*‐like* (*MtIPD3L*), function in early signaling pathways. *Defective in nitrogen fixation 4* (*MtDNF4*) and *MtDNF7* play roles in bacterial maturation but have no clear orthologous genes in soybean [4]. Toward identifying molecular mechanisms regulating SNF and its establishment in soybean, several studies have aimed to identify genes responsive to rhizobial infection through RNA‐seq. Three independent studies identified 6,099, 1,973, and 3,210 differentially expressed genes (DEGs) in soybean roots after rhizobium inoculation, respectively [9, 10, 11]. Although pathways regulating SNF can be identified through approaches such as gene ontology (GO) and Kyoto Encyclopedia of Genes and Genomes (KEGG) enrichment analysis, the expression of DEGs in a cell‐type‐non‐specific manner often hinders the identification of components that directly regulate SNF by diluting the cell‐type‐specific expression of candidate genes in bulk RNA‐seq data.

To establish a productive symbiosis, the host plant and its compatible rhizobium partner recognize each other through plant‐produced compounds such as flavonoids; pathogen‐derived Nod factors induced in response to recognition then initiate root hair deformation, the formation of an infection thread, and the establishment of a nodule meristem and primordium in the roots of host plants [12, 13, 14]. Genetic reprogramming therefore occurs in root‐hair cells, the epidermis, and the cortex, making these cells critical for symbiosis establishment, particularly during the early steps of the interaction. Bulk RNA‐seq profiling in entire roots may mask signals from small pools of cells of interest and confound later analysis. Single‐cell RNA‐seq (scRNA‐seq) is a practical method to access cell‐type‐specific transcriptome data and has been successfully applied to root cells. Since the first scRNA‐seq study of *Arabidopsis* roots [15], other studies have constructed developmental trajectories of root cells from several plant species [16, 17, 18, 19], revealing cell‐type‐specific as well as species‐specific regulatory networks. The rapid development of experimental procedures and computational pipelines, especially to extract cell‐type‐specific marker genes for tissues of interest, make scRNA‐seq an ideal approach for studying SNF establishment in soybean roots. To date, insights have been gained into nodule formation from 12–28 dpi [20, 21, 22]. However, despite these efforts, our understanding of the molecular mechanisms underlying early infection of soybean roots by rhizobia remains limited, and key regulatory genes have been seldom characterized.

Soybean forms determinate nodules that emerge from the middle and outer root cortex; these spherical nodules possess transiently active meristems and maintain a consistent developmental stage [23]. Once rhizobial‐produced Nod factors (NFs) are perceived by legume hosts, the common signal‐transduction pathway is triggered and initiates infection of root epidermal cells and organogenesis of nodules in cortical cells [24]. In soybean, the kinetics of nodule development are categorized into twenty stages (I–XX) [25]. Stages I–VII relate to the formation of young nodules, whereas stages IX through XX are characterized by nodule‐mass increase and cortex differentiation. Stages III through V appear to be the major control point in soybean nodulation [26]. Indeed, at 5 dpi, ∼35.4% of cells were in stages I or II, with another ∼51.2% in stages III–IV.

Here, to complement existing soybean studies and broaden the temporal resolution of symbiosis establishment, we aimed to identify cell‐type‐specific transcriptome signatures during the early stage of symbiosis establishment by scRNA‐seq at 4 dpi. By focusing particularly on epidermal cells and cortical cells, we identified cell‐cluster‐specific DEGs and key transcription‐factor genes in a regulatory network assembled from scRNA‐seq data. Informed by gene‐regulatory networks, functional analyses of a candidate gene and its physiological and developmental relevance showcase the feasibility of scRNA‐seq for discovering key pathways and genes participating in symbiosis establishment for subsequent nitrogen fixation.

## 2 Results

### 2.1 Development of a Soybean Root Single‐Cell Transcriptome Atlas at an Early Stage of Symbiosis Establishment

**FIGURE 1:**
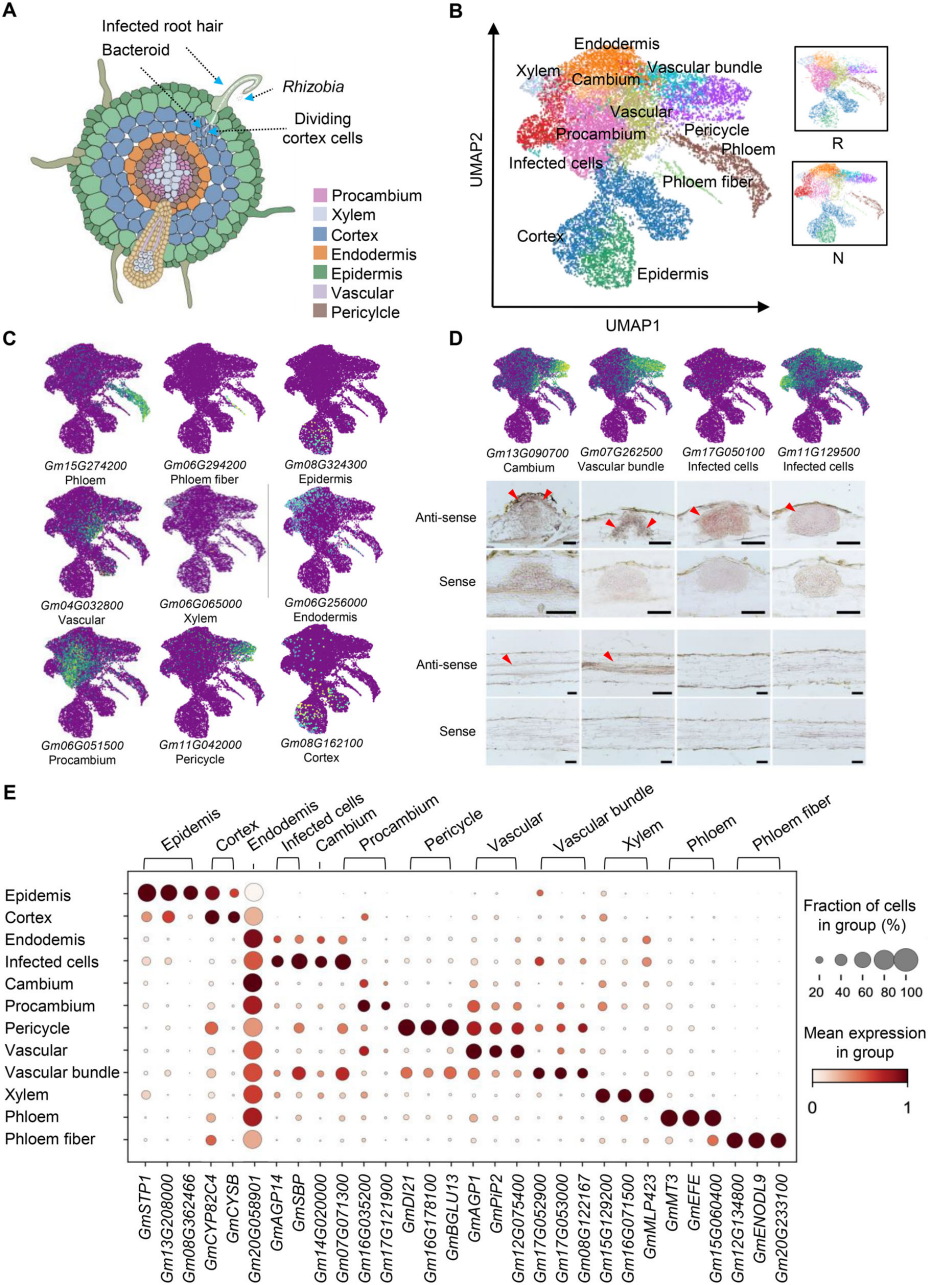
Establishment of a single‐nucleus transcriptome atlas of soybean roots after inoculation with *Bradyrhizobium japonicum*. (A) Cross‐section of a soybean root infected by *B. japonicum* at an early stage. (B) Uniform manifold approximation and projection (UMAP) plot showing 12 cell clusters obtained from the scRNA‐seq data of root cells challenged with *B. japonicum* (N group) or mock‐inoculated (R group), plotted together and separately (inset graphs). (C) UMAP plots showing the expression of known cell‐type‐specific marker genes used for the annotation of cell clusters. Each dot represents an individual cell. (D) Representative in situ hybridization of cell‐cluster‐enriched genes for the three putative clusters in young nodule and uninfected root. Scale bar: 100 µm. (E) Dotplot representation of the expression levels of root‐cell‐specific marker genes. The color gradient from white to red indicates the average expression in a given cell type.

To investigate cell type‐specific responses during early stages of symbiotic establishment, we inoculated soybean *cv*. Williams 82 roots at the early vegetative (V2) growth stages with *Bradyrhizobium japonicum* USDA110 (hereafter referred to as N group) or mock‐inoculated them with distilled water only (hereafter referred to as R group) and collected root samples at 4 days post inoculation (dpi). At this point, rhizobial infection threads have formed, and the nodule meristem and primordia are beginning to emerge (Figure 1A). Protoplasts were isolated from roots and loaded onto the microfluidic Chromium technology (10X Genomics) chip to generate scRNA‐seq libraries. After quality controls, the dataset encompassed expression data for 21,500 cells, with 9,161 cells from the N group and 12,339 cells from the R group. We obtained similar mapping statistics when aligning the clean trimmed reads to the *cv*. Williams 82 reference genome. On average, 77.45% of reads mapped confidently to the genome and 53.5% of reads aligned to the reference transcriptome, covering 40,255 (N group) and 39,421 genes (R group) and representing 47,095 genes in total. For each cell, we detected an average of 2,033 genes and 3,805 unique molecular identifiers (Table S1).

We conducted a dimensionality‐reduction step on the pooled dataset consisting of expression data from approximately 2,000 genes (average nGene), resulting in 12 distinct cell populations. We then visualized the 12‐cell cluster as a uniform manifold approximation and projection (UMAP) plot (Figure 1B). To annotate the tissue origin of cell clusters, we examined the expression pattern of 59 verified cell‐type‐specific marker genes (Table S2) [20, 21, 22]. We confidently assigned nine of the 12 cell clusters to a specific root tissue based on at least one marker gene being specifically expressed in those cells, leading to the annotation of phloem cells, phloem‐fiber, epidermal cells, endodermis, cortical cells, procambium cells, pericycle cells, vascular cells and xylem cells (Figure 1B, C; Figure S1). For the remaining three cell populations that lacked highly specific markers, infected cells were annotated based on the detection of this cluster in the N group and its absence in the R group (Figure 1B), together with the expression pattern of the known marker gene *Gm17G050100* [20] and a newly identified marker, *Gm11G129500*, which was validated by in situ hybridization in this study (Figure 1D). For the characterization of the remaining two clusters, we performed a homology search for the top‐10 identified cluster‐specific marker genes (Figure 1E; Table S3) against genes from *A. thaliana* [19] and *M. truncatula* [27, 28]. Through the integration of information about the biological relationships of these clusters with the cell clusters surrounding them in the UMAP plot and the expression of homologous marker genes, we assigned the two cell clusters as vascular bundle, and cambium cells, respectively (Figure 1B, C). In parallel, we identified cell‐type‐specific markers independently in the R and N datasets, and summarized the top‐20 highest‐specificity markers shared by both groups in Table S4. In situ hybridization analysis of these four markers in root and nodule tissues at 4 dpi confirmed their tissue‐specific expression patterns (Figure 1D). *Gm11G129500* and *Gm17G050100* were expressed exclusively in nodules, whereas the cambium marker *Gm13G090700* and the vascular marker *Gm07G262500* were also expressed in their respective tissue types within the uninfected root (Figure 1D, E; Table S3).

A UMAP‐based visual comparison of the R and N groups did not reveal any clear differences in the types of cells captured, except for the cluster identified as infected cells, which was enriched in the N group, representing 1,326 cells (12.9% of total) compared to only 61 (0.8% of total) in the R group (Figure 1B; Table S5). Given the very low abundance in the R group, these 61 cells are likely to be misclassified cells. Cortical cells were the most frequently captured, accounting for 19.4% of the captured cells, with comparable capture rates in the R and N groups at 20% and 18.8%, respectively; these were followed by procambium cells (16.3% of total) and vascular cells (11.1% of total). The least‐frequently‐captured cells were phloem‐fiber cells, representing only 1.4% (Table S5). Given that our sampling point coincided with the period of epidermis infection‐thread formation and progression to cortical layers to establish nodule primordia, another cell population of interest was epidermal cells, representing 7.5% of all captured cells; together with cortical cells and infected cells, these accounted for 33.7% of the total cell number. The adequate cellular population size provided the foundation for subsequent identification of cell‐type‐specific regulatory genes in early nodule formation, indicating suitable statistical power and reliability.

### 2.2 Cell‐Type‐Specific and Species‐Specific Responses Act during Early Nodule Development

Identifying differentially expressed genes (DEGs) between the mock and inoculated groups in each cell type could generate hypotheses for molecular mechanisms underlying early steps leading to symbiotic nitrogen fixation. Prior to DEG analysis between corresponding cell types in mock and inoculated roots, we performed an additional validation of the identities of the 61 R‐group cells initially classified as infected cells. Re‐clustering of cells in the R dataset alone showed that these 61 cells did not form a distinct infected‐cell cluster but instead scattered among other cell populations, indicating that these cells are likely not true infected cells alone (Figure S2A). By contrast, infected cells in the N group remained tightly clustered even when N dataset was analyzed alone (Figure S2B). Therefore, infected cells were excluded from the standard R sample based DEG analyses, and were subjected to a dedicated analysis as an independent cell population.

**FIGURE 2:**
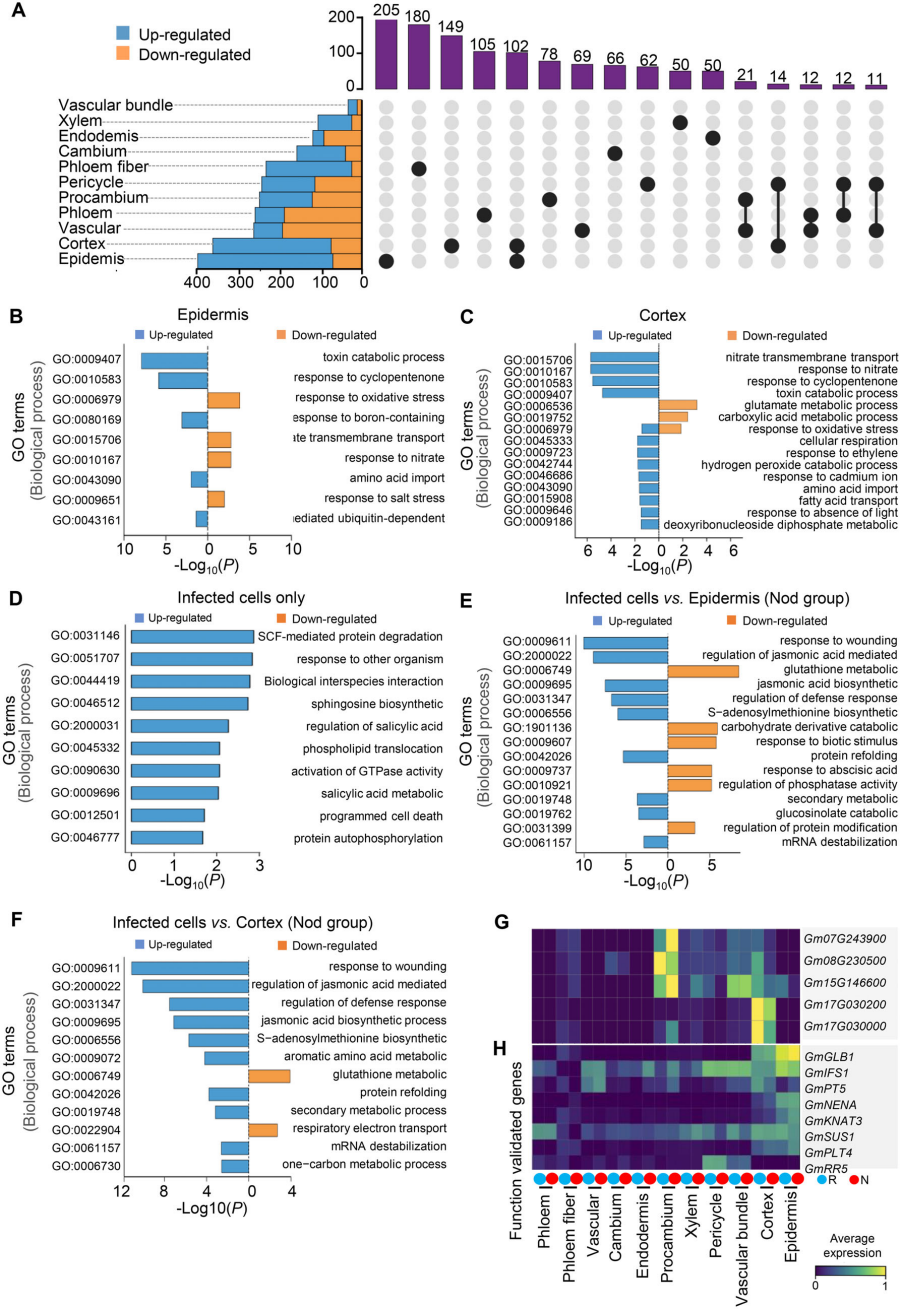
Identification of key genes and pathways involved in the initial establishment of symbiosis. (A) UpSetR plot showing the number of unique and shared differentially expressed genes (DEGs) among the 12 cell clusters. The numbers of upregulated and downregulated DEGs for each cell type is shown on the left. (B, C) GO enrichment analysis (Fisher's exact test) for biological processes of all DEGs between rhizobium‐inoculated and control groups in epidermal (B) and cortical (C) cells. (D) GO enrichment analysis for biological processes of genes specifically expressed in infected cells in the rhizobium‐inoculated group. (E, F) GO enrichment analysis for biological processes of DEGs between infected cells and epidermal (E) or cortical (F) cell populations in the rhizobium‐inoculated group. (G) Heatmap showing expression levels of genes in the GO term “response to biotic stimulus” in inoculated and control groups. (H) Heatmap showing expression levels of eight genes known to be required for symbiotic nitrogen fixation in legumes that were differentially expressed between the R and N groups.

A total of 1,522 unique DEGs were identified for the 11 remaining cell clusters shared between R and N groups (Figure 2A; Figure S3 and Table S6). A large portion of these DEGs was cell‐type‐specific, as 66.7% (1,015 genes) were differentially expressed exclusively in one cell type, whereas 20.8% (316 genes) were shared by two cell types, together accounting for 87.5% of all identified DEGs (Figure S3). Among 507 DEGs shared by at least two cell types, the direction of the change in expression for 137 (27.0%) of these was opposite between the two cell types (Table S6), suggesting that different cell types in soybeans roots make precise and flexible adjustments in gene‐expression responses to *B. japonicum* infection to ensure the successful establishment of symbiosis, as well as post‐infection control over rhizobia development and proliferation *in planta*. Among the 11 cell types, the bulk of DEGs were in epidermal cells (397 DEGs; 26.08%), followed by cortical cells (360 DEGs; 23.65%) (Figure 2A), representing two clusters containing cell types of greatest interest during early nodule formation. We further performed an intersection analysis between epidermis‐specific and cortex‐specific DEGs and identified 102 shared genes. Among them, 91 genes (89.2%) were consistently upregulated in both tissues, six genes (5.9%) were consistently downregulated, and only five genes showed opposite regulatory directions (Figure S4A). GO Biological Process enrichment of the 91 commonly upregulated genes indicated predominant enrichment in stress‐response and cellular homeostasis programs, such as processes related to redox homeostasis and ion balance (Figure S4B).

In parallel, GO enrichment of epidermis‐ and cortex‐specific DEGs suggested clear tissue‐level functional divergence during nodulation (Figure 2A, B; Table S7). Epidermis‐specific DEGs were mainly enriched in detoxification and proteostasis‐associated programs, with reduced representation of nitrate‐ and oxidative stress‐related terms, consistent with adaptive responses at the infection interface (Figure 2A). By contrast, cortex‐specific DEGs showed broader enrichment in transport, respiration, redox buffering, and hormone‐associated signaling, such as ethylene‐related pathway, together with selective attenuation of primary carbon and nitrogen metabolic processes (Figure 2B). Notably, nitrate‐related terms (GO:0010167, GO:0015706) displayed opposite enrichment directions between the two tissues, supporting a putative division of labor in which the epidermis prioritizes barrier and stress adaptation, whereas the cortex adopts a metabolically supportive state for nodule organogenesis.

Given the important roles of these cell types in the early stages of symbiosis establishment [4], we further examined the expression patterns of the top 10 most significantly enriched DEGs identified between the R and N groups in epidermal (Figure S5A) and cortical cells (Figure S5B). In epidermal cells, with the exception of the stress‐induced gene *Nod19* (*GmNod19‐19*, *Gm05G126900*), the remaining nine genes were downregulated. These genes were predominantly associated with GO terms related to ‘response to stress’, indicating that defense‐related pathways are partially suppressed in the epidermis to facilitate successful infection. In cortical cells, although the overall proportions of up‐ and downregulated DEGs were comparable to those in epidermal cells, the top‐10 most significant DEGs showed no clear directional bias, with five genes upregulated and five downregulated. Notably, five of these genes were also identified as DEGs in epidermal cells had similar expression trends in both tissues, while *GmNod19‐19* was also upregulated in cortical cells, further supporting that epidermal and cortical cells maintain distinct tissue‐specific programs during symbiosis establishment, yet still share certain conserved symbiotic regulatory mechanisms (Figure S4 and Table S6).

In pericycle and procambium cells, stress‐response‐related GO terms were enriched only among up‐regulated DEGs (Figure S6A, B). We checked the expression of five genes associated with ‘response to biotic stimulus’ across different cell types in the scRNA‐seq datasets. Without exception, all five were down‐regulated in either or both epidermal cells and cortical cells, while being up‐regulated in the surrounding cells wherein differential expression was seen (Figure 2G). The precise regulation of cell‐type‐specific gene expression appears to be critical for successfully establishing symbiosis and confining the scope of infection. Three of these five genes are involved in the abscisic acid (ABA) signaling pathway, suggesting an important role for this hormone in ensuring successful initial infection.

Since no corresponding infected‐cell cluster was detected in the control R group, we first resolved the cellular identity of the infected‐cell population in the N group using Decoupler and Scanpy. These approaches showed 69.8% concordance, supporting the robustness of the annotation trend. Integrating both results, we found that 15.69% of infected cells were epidermis‐like and 21.1% were cortex‐like, whereas the remaining cells were classified as mixed, indicating substantial heterogeneity in their origin (Figure S2C, D). Based on this heterogeneity, we conducted differential‐expression analyses at three levels: infected cells versus all other cell types, infected cells versus epidermal cells, and infected cells versus cortical cells (Table S8). GO enrichment analyses across the three comparison levels further clarified the biological identity of infected cells. Relative to all other cell types, infected cell‐specific genes were predominantly enriched in processes associated with interspecies interaction, response to other organisms, proteasome‐dependent protein turnover, membrane lipid remodeling, and salicylic acid‐related signaling, indicating that infected cells represent a specialized host‐microbe interaction state (Figure 2D). Compared with epidermal cells, infected cells showed stronger enrichment in wounding‐, jasmonate‐, and defense‐regulatory pathways, but lower enrichment in glutathione metabolism and broader biotic‐stimulus responses, suggesting a shift from general frontline stress buffering toward a more specialized infection‐associated program (Figure 2E). In comparison with cortical cells, infected cells similarly displayed enhanced wound‐, jasmonate‐, and defense‐associated processes, but reduced enrichment of respiratory electron transport and glutathione metabolism, indicating that they are also distinct from the metabolically supportive cortical state (Figure 2F). Together, these results support the view that infected cells occupy a unique transitional symbiotic state, molecularly distinct from both epidermal and cortical cells.

We also examined expression patterns of soybean orthologs of functionally validated genes involved in symbiotic nitrogen fixation (SNF) in other legumes [4]. In our scRNA‐seq data, we detected 37 genes expressed at least in 10 cells (Figures 2H; Figures S7 and S8). Most genes showed cell‐type‐specific expression, with eight being differentially expressed between the R and N groups (Figure 2H; Figure S8). Using expression in the R group as a reference, among these eight DEGs, *GLOBIN 1* (*GmGLB1*; *Gm11G121800*) was up‐regulated in epidermal and cortical cells. The nucleoporin gene *GmNENA* (*Gm13G155800*), *KNOTTED1‐LIKE HOMEOBOX GENE 3* (*GmKNAT3*; *Gm14G112400*), and *PLETHORA 4* (*GmPLT4*; *Gm01G022500*) were up‐regulated, but *SUCROSE SYNTHASE 1* (*GmSUS1*; *Gm09G073600*) was down‐regulated in epidermal cells. *PHOSPHATE TRANSPORTER 5* (*GmPT5*; *Gm03G162800*) was down‐regulated in cortical cells and up‐regulated in procambium cells, but *ISOFLAVONE SYNTHASE 1* (*GmIFS1*; *Gm07G202300*) was up‐regulated in cortical cells (Figure 2H). For the five genes up‐regulated in either epidermal or cortical cells, *GmIFS1* and the orthologs of *GmNENA* in *L. japonicus* function in early signaling, and the ortholog of *GmGLB1* in *L. japonicus* is essential to ensure successful infection [29]. The orthologs of *GmPLT4* and *GmKNAT3* in alfalfa (*M. sativa*) are critical for nodule organogenesis [30], and the cortex‐specific down‐regulated gene *GmPT5* and the *L. japonicus* ortholog of the epidermal‐cell‐specific down‐regulated gene *GmSUS1* are both involved in nodule sugar metabolism and transport [31]. Not surprisingly, many genes that showed strong differential expression between the R and N groups in our data have not been reported in *L. japonicus* or alfalfa. Conversely, many orthologs of signal‐perception genes reported in *L. japonicus* and alfalfa either were not detected in our data or did not show differential expression (Figure S7 and Table S6). This discrepancy may partly reflect technical differences between bulk RNA‐seq and snRNA‐seq, but may also indicate genuine divergence in the regulatory mechanisms governing symbiosis establishment across legume hosts.

To further compare rhizobial responses during symbiotic nitrogen fixation across species and identify potential shared regulatory pathways among nodulating species, we collected published single‐cell RNA‐seq datasets from soybean [22, 32], *Medicago truncatula* [33], and *Lotus japonicus* [34]. Because strong batch effects prevented reliable cross‐dataset integration, we performed GO enrichment analysis separately for each dataset and focus on cortical cells to identify both shared and species‐specific pathways. The two soybean cortex stages share several core response modules, including ethylene signaling (GO:0009723), carboxylic‐acid‐related metabolism (GO:0019752, GO:0046394), and redox‐associated processes (GO:0006979), indicating a conserved baseline program during nodulation (Figure 2C; Figure S9D). However, in cross‐species comparisons, we observed substantial differences in enrichment profiles. These discrepancies may reflect species‐specific regulatory programs, but they may also arise from non‐matched sampling time points across datasets. Despite this divergence, *Medicago* and *Lotus* shared clear enrichment of cell wall remodeling processes (GO:0042546, GO:0030244, GO:0016998), involving in cell wall organization/modification, primary cell wall biogenesis, and cellulose biosynthesis, whereas soybean and Lotus shared redox homeostasis‐related pathways (GO:0006979). Taken together, these results indicate partial conservation of nodulation‐associated programs across species and nodule types, while the relative pathway weights and regulatory emphases remain species dependent.

### 2.3 Gene Regulatory Network Construction During Early Symbiosis Establishment

**FIGURE 3:**
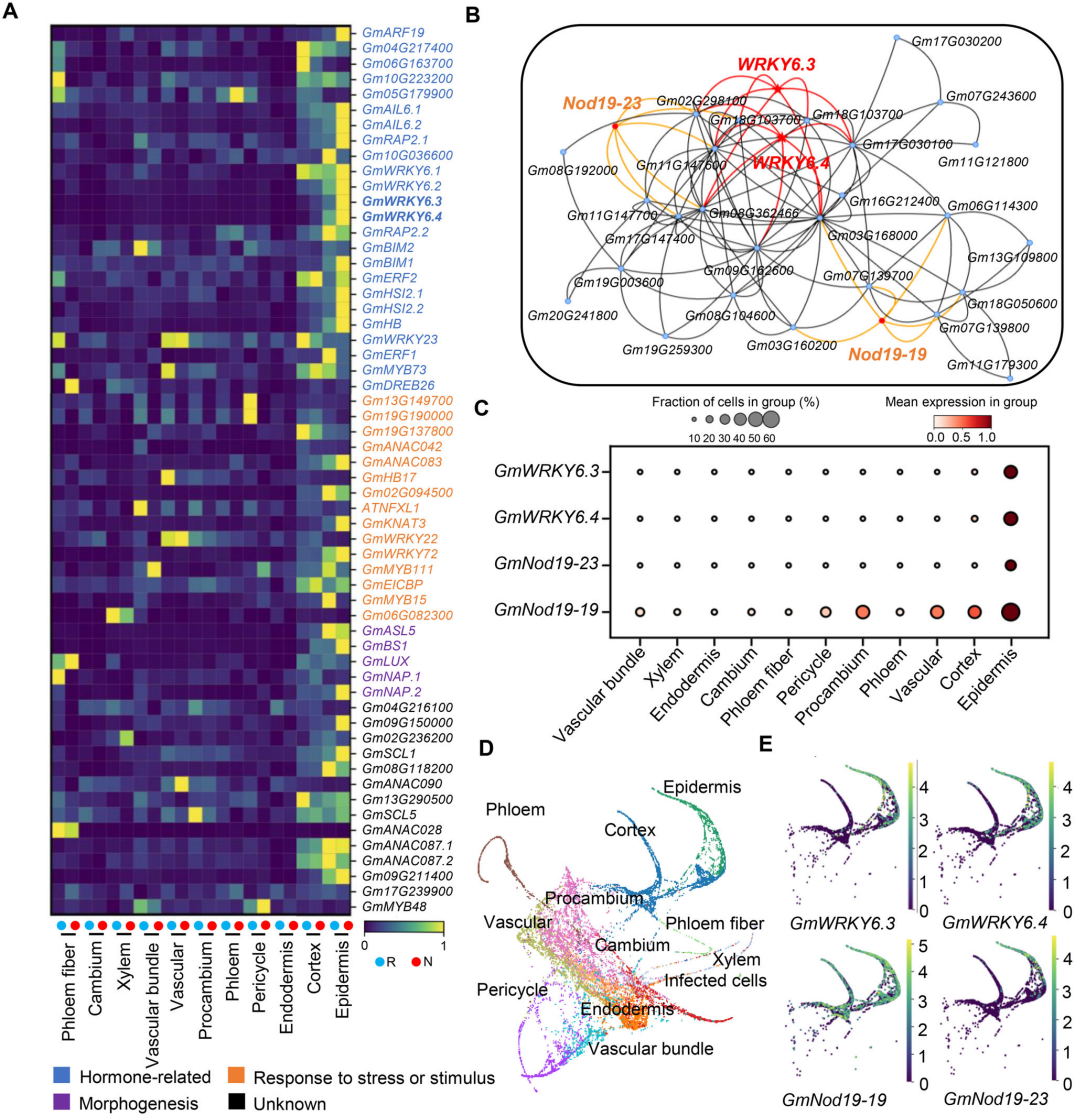
Construction and analysis of gene co‐expression networks. (A) Heatmap representation of the expression levels of 58 cell‐type‐specific transcription‐factor genes differentially expressed between the R and N groups. Genes are shown in rows and cell clusters in columns. Genes encoding transcription factors are colored based on their associated pathways. (B) A gene regulatory network (GRN) inferred for *GmNod19‐19* and *GmNOD19‐23* (shown in orange) from scRNA‐seq data using the RegDiffusion algorithm with the cutoff *k* = 15. (C) Single‐cell‐type expression of *GmWRKY6.3*, *GmWRKY6.4*, *GmNOD19‐19*, and *GmNod19‐23*. (D, E) Inferred cell‐type‐specific developmental trajectory using partition‐based graph abstraction (PAGA; D) and visualization of expression levels for *GmWRKY6.3*, *GmWRKY6.4*, *GmNod19‐19*, and *GmNOD19‐23*. (E) Hairy‐root‐mediated analysis of *GmWRKY6.3* and *GmWRKY6.4* knockout effects on *GmNod19‐19* and *GmNod19‐23* expression at 4 dpi. (F) Effect of exogenous application of the ethylene precursor (ACC) and inhibitor (AVG) on the expression of *GmWRKY6.3*, *GmWRKY6.4*, *GmNod19‐19* and *GmNod19‐23* at 4 dpi. Data are presented as mean ± SD (n = 10). Treatments sharing the same letter within each panel do not differ significantly (one‐way ANOVA with Tukey's HSD post‐hoc test, *p* < 0.05).

Integrating DEGs into a gene regulatory network (GRN) can be helpful toward understanding the underlying regulatory circuits that drive cellular responses [35]. Using DEGs encoding transcription factors as the framework owing to their role in gene regulation and known activities in symbiosis establishment, we constructed a root GRN for the early stages of rhizobium infection using diffusion models, which are widely used in generative artificial intelligence (Figure S10). Among the 1,715 DEGs obtained above, including 193 genes specifically identified in infected cells and 1,522 DEGs identified from the other 11 cell clusters, 58 are predicted to encode transcription factors (Figure 3A; Table S9). Based on functional annotations, the most common group of transcription factors encoded by these DEGs are those containing an ethylene‐response factor (ERF) domain (n = 9 DEGs; 15.6%), followed by those containing NAC and WRKY domains, with eight each (13.8%), and by those with other domains such as MYB and basic helix‐loop‐helix (bHLH) domains (Table S9). We sought to understand the physiological processes controlled by these different transcription‐factor groups based on functional annotation of respective biological‐process GO terms. Apart from 14 genes for which there was no specific annotation information, 24 of the remaining 44 genes are associated with hormone‐related pathways, and 16 of the 24 are involved in the ethylene pathway. Of the other 20 genes, 15 can be categorized into pathways related to ‘response to stress and environmental stimulus’, with the remaining five involved in plant morphogenesis, growth, and development (Figure 3A; Table S9). Using all transcription factors identified at the whole‐genome level as the background, we further performed enrichment analysis on the functionally annotated transcription factors in these three categories and found that only ethylene‐responsive transcription factors showed statistically significant over‐representation (*p* = 7.830461e‐12). These analyses suggest a central role for hormones, particularly ethylene, in early infection by rhizobia and the successful symbiotic establishment.

Using 6,519 highly variable genes identified by Scanpy, we generated a GRN comprising 430 genes associated with 58 transcription factors, of which 309 were DEGs (18% of the 1,715 DEGs). Relatively stringent parameters were used to construct this GRN (Table S9), and additional DEGs could be incorporated by relaxing the thresholds. The inferred network consisted of a main GRN and two smaller branches. The main GRN was organized around an ethylene‐responsive core that contained most transcription‐factor nodes and had extensive connections with other hormone‐associated components, including jasmonate (JA), salicylic acid (SA), and brassinosteroids (Figure S10). A second branch was centered on an auxin‐related regulatory module containing *AUXIN‐RESPONSE FACTOR 19* (*GmARF19*) and genes implicated in growth and development, including *GmNAP1* (*Gm11G136600*) and *GmLUX* (*Gm07G229100*; Figure S10). The third branch was linked to hypoxia‐related responses (Figure S10), containing three wound‐responsive genes (*Gm13G301700*, *Gm12G218300*, and *Gm12G200500*). Coordinated hormone regulation is therefore essential for orchestrating early infection and nodule initiation in soybean. We then performed a hormone‐crosstalk analysis by assigning hormone‐related keywords in gene annotations and GO biological‐process terms, and quantified crosstalk as the co‐occurrence of two hormone categories within the same DEG entry. Consistent with constructed GRNs, ethylene–JA and ethylene–SA were the most and third‐most frequent interaction, while auxin–JA was the second‐most frequent item (Figure S11). Crosstalk among ethylene, JA, and SA may fine‐tune infection progression while restraining excessive immune activation, whereas auxin‐associated crosstalk may help integrate developmental programs with infection‐triggered cues.

Given the importance of ethylene in symbiosis establishment as identified above, we further examined transcription factors and DEGs with major differentials within the GRN. *GmNod19‐19*, which was the only up‐regulated DEG identified among the top‐10 most differentially expressed genes in epidermal cells, appeared to be under the control of the transcription factors *GmWRKY6.3* (*Gm18G092200*) and *GmWRKY6.4* (*Gm08G320200*) in the network, both of which are involved in ethylene‐activated signaling pathways based on their annotations. *GmNod19‐23* (*Gm15G131400*) is another epidermal‐cell‐specific DEG belonging to the same family as *GmNod19‐19*, the expression of which was induced in epidermal cells and is also under the control of *GmWRKY6.3* and *GmWRKY6.4* within the GRN (Figure 3B). We then analyzed the expression patterns and differentiation trajectories of these four genes across different cell types (Figure 3C–E; Figure S12). At the experimental time points examined, *GmWRKY6.3* and *GmWRKY6.4* expression was restricted to epidermal cells (Figure 3C). *GmNod19‐19* was highly expressed in epidermal cells but also showed relatively high expression levels in cortical, procambium, and vascular cells. A cell‐differentiation‐trajectory analysis of *GmNod19‐19* indicated that although it is expressed at relatively low levels in cortical cells, it was expressed in almost all cortical cells in various stages of differentiation (Figure 3D). By contrast, *GmNod19‐23* expression was highly specific to epidermal cells, being expressed only in highly differentiated epidermal cells located at the tip of the differentiation trajectory (Figure 3D, E) and showing a trajectory more similar to those of *GmWRKY6.3* and *GmWRKY6.4* than to *GmNod19‐19* (Figure 3E).

The cell‐type‐specific expression of these four genes was also confirmed using SnRNA‐seq data generated from soybean roots at 12 dpi and 15 dpi (Figure S9C, D) [22, 32]. We further examined the spatial transcriptomic data from Liu et al. [22]. For *GmWRKY6.3*, *GmWRKY6.4*, and *GmNod19‐23*, spatial signals were either below the detection threshold or lacked sufficient resolution for reliable cell‐type‐level assignment. *GmNod19‐19* was detected in nodule tissue at both 12 and 15 dpi, with expression predominantly enriched in the nodule rather than the root (Figure S9F), while a clear preference for specific nodule zones could not be resolved at the available resolution, the convergence of ScRNA‐seq and spatial data strengthens the evidence for its nodule‐associated expression. The tissue‐specific expression of these four genes was also examined in cross‐species datasets [33, 34]. Their homologs in *M. truncatula* showed comparable expression patterns (Figure S9A), whereas in *L. japonicus*, the expression patterns of the homologous genes differed substantially (Figure S9B).

*GmNod19‐23* is predicted to be a target gene of *gma*‐*miR398e* (Figure S7), which is a key regulator of plant development and stress responses [36]. It is involved in responses to ethylene [37], ABA [36], JA [38] and SA [39]. Typical *cis*‐elements in the promoter region of *miR398e* genes include a W‐box, which is essential for the binding of WRKY transcription factors and responsiveness to stress [36, 40]. We also performed a multiple sequence alignment of *GmNod19‐23* and predicted miR398s in soybean (Figure S13A, B). Here, by combining a bioinformatic analysis of *GmNod19‐23*, a cell‐type‐specific GO‐enrichment analysis, and a GRN, we propose that gma‐*mirR398* may be involved in communication among ethylene and other hormone signaling pathways, coordinately regulating the plant immune response and nodule formation through GmWRKY6.3/6.4 and their targets *GmNod19‐19/23*.

### 2.4 Validation of *GmNodl9‐19/23* Regulation by GmWRKY6.3/6.4

**FIGURE 4:**
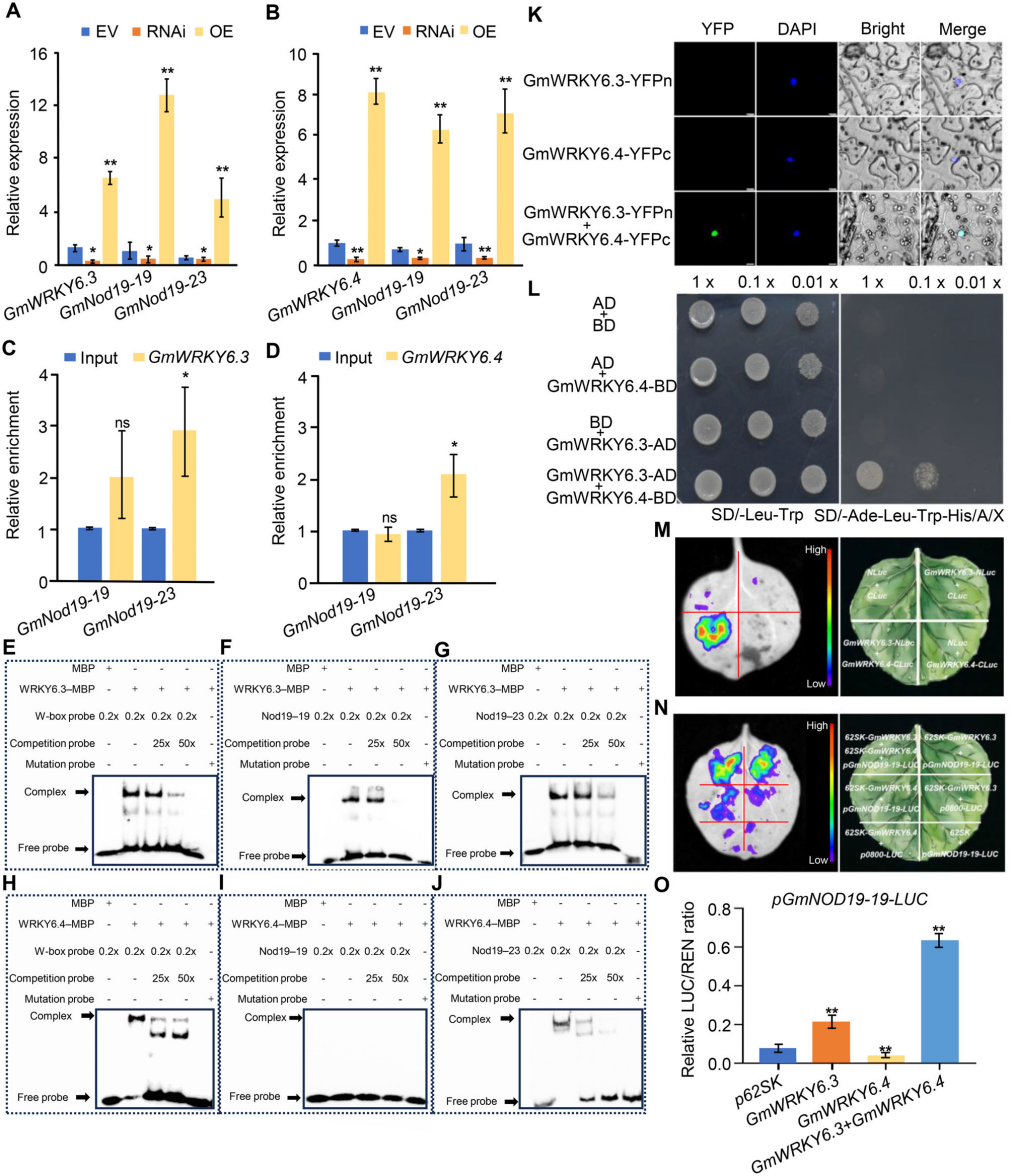
GmWRKY6.3/6.4 transcription factors bind to and activate select *GmNod19* promoters. (A, B) Relative transcript levels of *GmNod19‐19* and *GmNod19‐23* in hairy roots at 4 dpi in *GmWRKY6.3* (A) or *GmWRKY6.4* (B) overexpression (OE) and RNA interference (RNAi) lines compared with empty‐vector (EV) controls. (C, D) ChIP–qPCR analysis using anti‐GFP antibody assaying enrichment of GmWRKY6.3–GFP (C) and GmWRKY6.4–GFP (D) on *GmNod19‐19* and *GmNod19‐23* promoters. (E–J) EMSA of GmWRKY6.3–MBP (E–G) and GmWRKY6.4–MBP (H–J) binding in vitro to W‐box‐containing probes derived from the promoters of *GmNod19‐19* (F, I) and *GmNod19‐23* (G, J). (K) Bimolecular fluorescence complementation (BiFC) assay examining the physical interaction between GmWRKY6.3 and GmWRKY6.4 in transiently transgenic *N. benthamiana* leaves. Scale bar: 10 µm. (L) Yeast two‐hybrid assay examining the physical interaction between GmWRKY6.3 and GmWRKY6.4. (M) Luciferase complementation imaging (LCI) assay examining the physical interaction between GmWRKY6.3 and GmWRKY6.4 in transiently transgenic N. benthamiana leaves. (N, O) Representative chemiluminescence images (N) and relative LUC/REN ratios (O) from dual‐luciferase reporter assays evaluating the transcriptional activation of the GmNod19‐19 promoter by GmWRKY6.3 and GmWRKY6.4. Data are presented as mean ± SD (n = 3). Statistical significance was determined by Kruskal–Wallis test (*p* < 0.05).

Guided by the single‐cell GRN analysis (Figure 3B), we sought to experimentally validate a putative regulatory circuit comprising the transcription factors GmWRKY6.3 and GmWRKY6.4 and their putative downstream targets *GmNod19‐19* and *GmNod19‐23*. In transgenic soybean hairy roots at 4 dpi, over‐expression (OE) of *GmWRKY6.3* or *GmWRKY6.4* resulted in up‐regulation of both *GmNod19‐19* and *GmNod19‐23* relative to the empty‐vector control (EV), whereas RNAi‐mediated knockdown of either gene resulted in down‐regulation of both *GmNod19* genes (Figure 4A, B). These genetic experiments position GmWRKY6.3 and GmWRKY6.4 as positive regulators of *GmNod19‐19* and *GmNod19‐23* during early rhizobial infection.

ChIP–qPCR revealed robust enrichment of GmWRKY6.3–GFP at the promoters of both *GmNod19‐19* and *GmNod19‐23*, while GmWRKY6.4–GFP was enriched specifically at the *GmNod19‐23* promoter but not detected at *GmNod19‐19* (Figure 4C, D). These binding patterns support a model in which GmWRKY6.3 directly activates both *GmNod19* targets, whereas GmWRKY6.4 preferentially engages *GmNod19‐23*. In EMSAs, GmWRKY6.3–MBP could bind in vitro to probes containing W boxes derived from the promoter regions of *GmNod19‐19* and *GmNod19‐23* (Figure 4E–G), while GmWRKY6.4–MBP bound a probe containing the W box from *GmNod19‐23* but not from the *GmNod19‐19* promoter (Figure 4H–J). Competition and shift patterns were concordant with the ChIP–qPCR results (Figure 4C, D), collectively demonstrating direct, motif‐dependent interactions.

As our ChIP‐qPCR and EMSA assays showed that GmWRKY6.4 does not directly bind the *GmNod19‐19* promoter, whereas RNAi‐mediated silencing of *GmWRKY6.4* still markedly reduced *GmNod19‐19* expression (Figure 4B), we reasoned that GmWRKY6.4 may regulate *GmNod19‐19* indirectly through GmWRKY6.3. In support of this hypothesis, BiFC showed that co‐expression of GmWRKY6.3‐nYFP and GmWRKY6.4‐cYFP generated a clear nuclear YFP signal, indicating that the two proteins can physically interact in planta (Figure 4K). This interaction was further supported by yeast two‐hybrid analysis (Figure 4L) and luciferase complementation imaging (Figure 4M). Consistent with their physical association, dual‐luciferase reporter assays showed that co‐expression of GmWRKY6.3 and GmWRKY6.4 activated the *GmNod19‐19* promoter more strongly than GmWRKY6.3 alone (Figure 4N, O). Together with the co‐expression, ChIP–qPCR, and EMSA data, these results support a model in which GmWRKY6.3 directly activates both *GmNod19‐19* and *GmNod19‐23*, whereas GmWRKY6.4 directly regulates *GmNod19‐23* but promotes *GmNod19‐19* expression indirectly through its interaction with GmWRKY6.3.

### 2.5 GmWRKY6.3/6.4 And GmNodl9‐19/23 Act Principally in Infection‐Thread Formation

**FIGURE 5:**
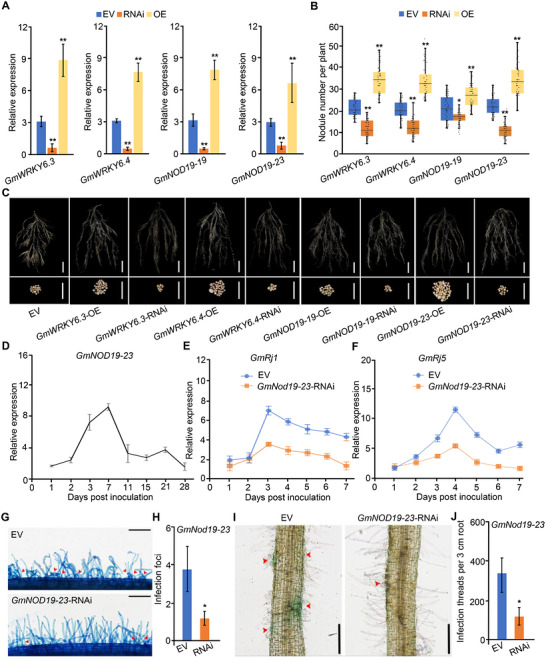
Manipulation of *WRKY6.3/6.4 or GmNod19‐19/23* expression alters root nodulation. (A) qRT–PCR of target gene expression in transgenic hairy roots at 4 dpi. (B, C) Nodule number quantification (B) and representative phenotypes (C) of transgenic hairy roots with the empty‐vector control, over‐expression or RNAi against *GmWRKY6.3/6.4* or *GmNod19‐19/23*. Scale bar: 3 cm. (D) Time‐course expression profile of *GmNod19‐23* after rhizobial inoculation. (E, F) Expression analysis of infection thread markers genes *GmRj1* (E) and *GmRj5* (F) in EV and *GmNod19/23* RNAi roots at 1–7 dpi. (G, H) Phenotypic characterization (G) and quantification of infection foci (H) in EV and *GmNod19/23* RNAi roots. Scale bar: 200 µm. (I, J) Phenotypic characterization (I) and quantification of infection threads (J) in EV and *GmNod19‐23‐RNAi* roots. Scale bar: 3 cm. Data are presented as mean ± SD (n = 10 biological replicates). Statistical significance was determined by two‐tailed Student's t‐test (*p < 0.05, **p < 0.01). EV, empty vector; OE, over‐expression.

To understand the function *in planta* of this WRKY‐dependent genetic circuit in the context of soybean nodulation, transgenic hairy root lines were developed with over‐expression or knock‐down cassettes for each of *GmWRKY6.3*, *GmWRKY6.4*, *GmNod19‐19* and *GmNod19‐23* (Figure 5A). Manipulation of all four genes resulted in phenotypes indicative of roles as positive regulators of root nodulation. Both sets of transgenic lines displayed opposing, dramatic phenotypes, with over‐expression plants forming statistically significantly more root nodules than EV controls, with RNAi resulting in a marked reduction in nodule number per plant. Manipulation of *GmNod19‐23* conferred the most pronounced effect (Figure 5B, C).

Using *GmNod19‐23* as representative of this regulatory circuit, we sought to pinpoint at which stage(s) in the nodulation pathway these genes function. First, a time‐course expression profile of *GmNod19‐23* was obtained, in which its transcript levels rose sharply by 2 dpi, and gradually declined by 7 dpi (Figure 5D), suggesting it functions during the early, infection‐centered stages of symbiosis establishment. Genetic markers of infection‐thread formation *GmRj1* and *GmRj5* showed altered expression in RNAi lines (Figure 5E, F). In contrast, markers for pre‐infection signaling (*GmIFS1*, *GmIFS2, GmNSP1*) and nodule‐primordium development (*GmNINa*, *GmENOD40*, *GmRR1d*) showed similar expression patterns between EV controls and RNAi plants (Figure S14). This marker profile suggested a specific involvement of at least *GmNod19‐23* in infection‐thread formation, rather than upstream signal recognition or nodule organogenesis. Quantification of infection events on *GmNod19‐23*‐silenced roots confirmed this inference, with infection foci (Figure 5G, H) and infection threads (Figure 5I, J) markedly reduced compared with EV controls. Taken together, these results establish that the regulatory circuit involving *GmWRKY6.3*/*6.4* and downstream their respective targets *GmNod19‐19/23* acts primarily during the early infection phase to promote infection‐thread formation and rhizobial invasion, thereby contributing to final root‐nodule number.

### 2.6 GmWRKY6.3/6.4 And GmNodl9‐19/23 Are Responsive to Ethylene

**FIGURE 6:**
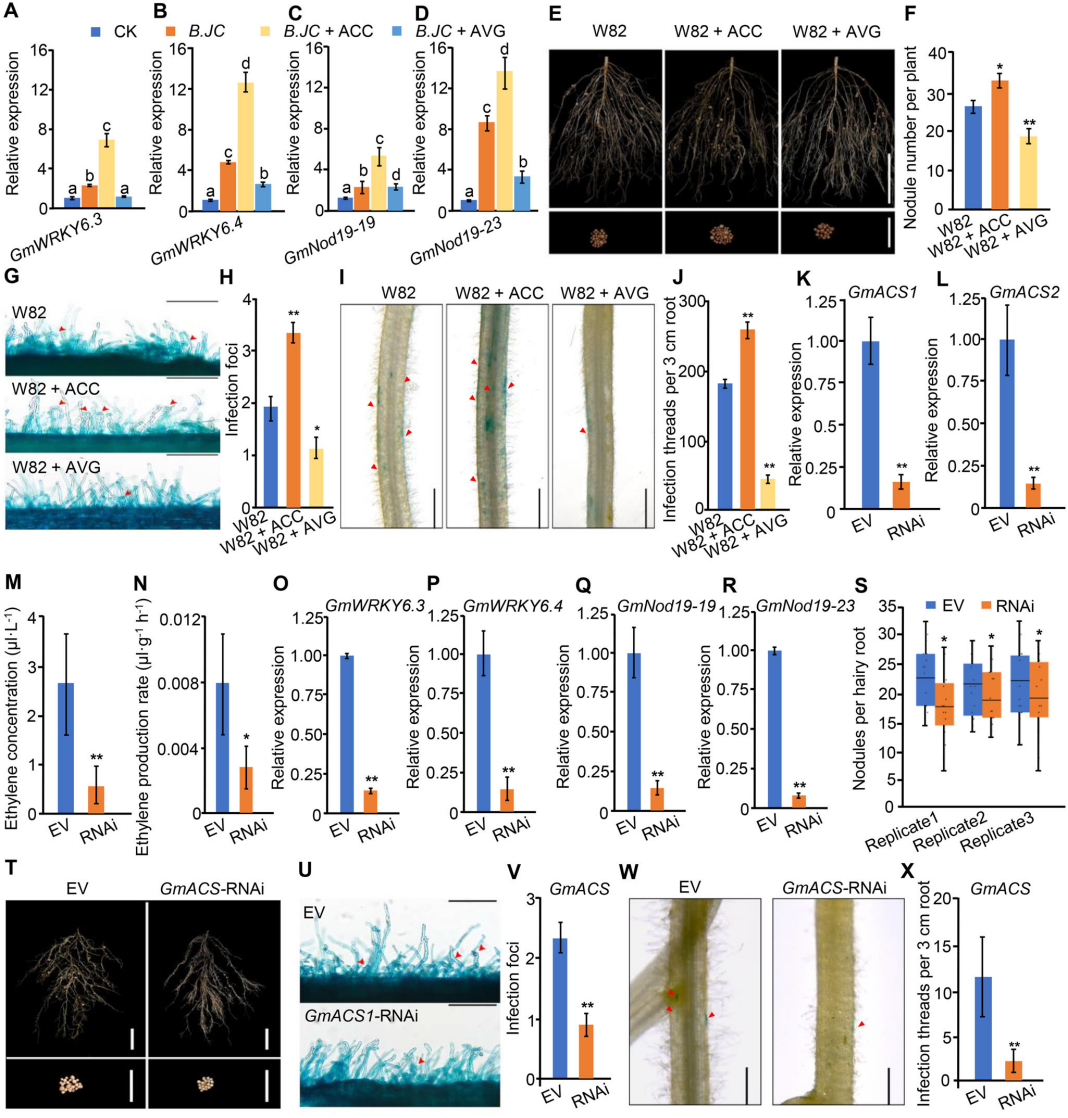
The genetic circuit comprising *GmWRKY6.3*/*6.4* and their targets *GmNod19‐19/23* is responsive to ethylene in soybean root nodulation. (A–D) qRT–PCR analysis in hairy roots at 4 dpi following pharmacological manipulation of ethylene signaling. (E, F) Phenotypic characterization (E) and nodule number quantification (F) at 28 dpi in W82 roots, ACC‐treated W82 roots, and AVG‐treated W82 roots. Scale bar: 8 cm. (G, H) Phenotypic characterization (G) and quantification of infection foci (H) in W82 roots, ACC‐treated W82 roots, and AVG‐treated W82 roots. Scale bar: 200 µm. (I, J) Phenotypic characterization (I) and quantification of infection threads (J) in W82 roots, ACC‐treated W82 roots, and AVG‐treated W82 roots. Scale bar: 3 cm. (K, L) qRT–PCR validation of RNAi‐mediated knockdown of the two root‐abundant ACC synthase genes *GmACS1* and *GmACS2* in hairy roots at 4 dpi. (M, N) Ethylene levels (M) and ethylene production rates (N) in empty‐vector (EV) controls and *GmACS1*/*2* dual RNAi roots. (O–R) qRT–PCR analysis of *GmWRKY6.3*, *GmWRKY6.4*, *GmNod19‐19*, and *GmNod19‐23* expression in EV controls and *GmACS1*/*2* dual RNAi roots at 4 dpi. (S, T) Nodule number quantification (S) and phenotypic characterization (T) at 28 dpi in EV controls and *GmACS1*/*2* dual RNAi roots across three experimental replicates. Scale bar: 3 cm. (U, V) Phenotypic characterization (U) and quantification of infection foci (V) in EV and *GmACS* RNAi roots. Scale bar: 200 µm. (W, X) Phenotypic characterization (W) and quantification of infection threads (X) in EV and *GmACS* RNAi roots. Scale bar: 3 cm. Data are presented as mean ± SD (n = 10 for qRT–PCR panels; n = 20 per treatment for nodulation phenotyping; n = 11 roots for infection foci/thread counts). Statistical significance was determined using two‐tailed Student's t‐test (*p < 0.05, **p < 0.01). EV, empty vector.

Based upon functional annotation, both *GmWRKY6*.*3* and *GmWRKY6.4* are involved in ethylene‐activated signaling pathways (Table S9), and the ethylene pathway is enriched in the GRNs acting during nodulation (Figure S10). We sought to experimentally determine whether ethylene signaling contributes to regulating the expression of *GmWRKY6.3/6.4* and their respective *GmNod19* downstream targets. In wild‐type Williams 82 plants, when inoculated roots were treated with the ethylene precursor 1‐aminocyclopropane‐1‐carboxylic acid (ACC), all of *GmWRKY6.3*/*6.4* and *GmNod19‐19*/*23* were up‐regulated compared with mock‐treated controls at 4 dpi (Figure 6A–D). Conversely, application of the ethylene biosynthesis inhibitor aminoethoxyvinylglycine (AVG) suppressed expression of these genes relative to controls at 4 dpi, suggesting that ethylene positively regulates this pathway. We further examined infection foci and infection thread formation in roots of both control and the 4 dpi treatment groups, and also quantified nodule numbers at 28 dpi. In Williams 82, compared with the mock treatment, AVG treatment significantly increased the number of infection foci, infection threads, and nodules, whereas ACC treatment had the opposite effect (Figure 6E‐J), indicating that ethylene modulates nodulation by affecting infection thread formation.

To test whether changes in endogenous ethylene levels contribute in vivo to modulating expression of *GmWRKY6.3/6.4* and *GmNod19‐19/23*, we sought to genetically down‐regulate ethylene biosynthesis in roots. Of the 20 annotated ACC synthase (*ACS*) genes in the Williams 82 reference genome, *GmACS1* (*Gm05G211700*) and *GmACS2* (*Gm08G018000)* are highly expressed in roots (Figure S15). Our single‐cell dataset showed that both *GmACS2* and *GmACS2* are expressed in infected cells, but *GmACS2* is statistically significant enriched (*p* = 9.004e‐26) in infected cells compared with other cell types (Figure S16). Both were targeted for simultaneous down‐regulation by RNAi in hairy roots (Figure 6K, L), and *ACS* down‐regulation was accompanied by a marked reduction in ethylene steady‐state levels and ethylene production in roots (Figure 6M, N). In dual *ACS* RNAi lines, transcript levels of *GmWRKY6.3*/*6.4* and *GmNod19‐19*/*23* were statistically significantly lower than in EV controls at 4 dpi (Figure 6O–R), establishing a link between root ethylene biosynthesis and expression levels of this circuit. RNAi of *ACS* significantly decreased the number of infection foci, infection threads, and nodules (Figure 6T‐X). However, at 28 dpi, *ACS* RNAi plants had fewer root nodules than EV controls, and the magnitude of this reduction was less pronounced than that observed with single‐gene knock‐down of individual targets (Figure 6S, T). This more modest nodulation phenotype likely reflects the broader pleiotropy of ethylene signaling, a hormone that impacts multiple developmental and defense pathways [41]. To further confirm the connection between the ethylene pathway and the GmWRKY6.3/6.4 regulatory module, we selected five representative ethylene pathway genes (*GmETR1a*, *GmACS1*, *GmACS2*, *GmEIN2a*, *GmEIN3b*) and performed correlation analysis of their expression with *GmWRKY6.3/6.4* under different treatments and time points. Except for GmEIN3b, *GmWRKY6.3/6.4* and the other ethylene pathway genes showed strong positive correlations (r > 0.6), further supporting that ethylene likely influences nodulation through the regulation of *WRKY6.3/6.4* (Figure S17).

To investigate whether the effect of ethylene on nodulation phenotypes is broadly conserved in soybean, we extended our analysis beyond Williams 82 to include two additional major cultivars, Qi Huang 34 (QH34) and Dong Nong 50 (DN50) with distinct genetically and agronomically backgrounds. Results with QH34 were consistent with those observed in Williams 82, whereas in DN50, the nodulation phenotype was largely insensitive to ethylene treatment (Figure S18). Collectively, these results establish ethylene as a key upstream regulator of *GmWRKY6.3/6.4* and their targets *GmNod19‐19/23* for control of early rhizobial infection. However, the effect of ethylene on soybean nodulation is apparently manifested in a cultivar‐dependent manner.

### 2.7 Evolution of the Stress Up‐Regulated Nod19 Family

**FIGURE 7:**
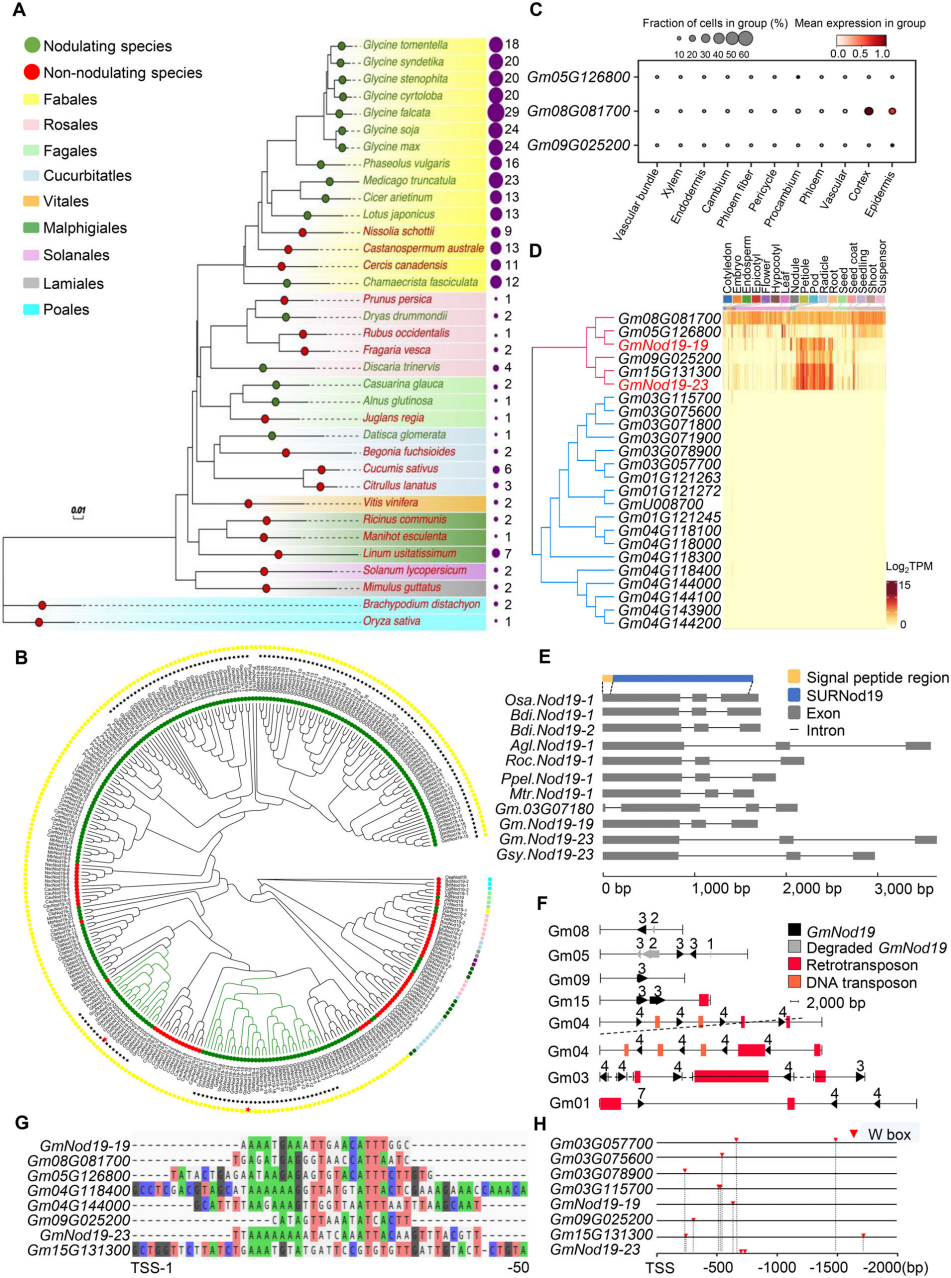
Phylogenetic profiling of the *Stress up‐regulated Nod19* superfamily and investigation of gene expansion in soybean. (A) Phylogenetic tree displaying the number of genes belonging to the *Nod19* family in each plant species. Green and red dots indicate nitrogen‐fixing and non‐nitrogen‐fixing species, respectively. The different plant orders are shown with different colored shading. Scale bar: 0.01 substitution per site. (B) Neighbor‐joining tree of 307 Nod proteins from 35 species. In the innermost circle, green and red dots indicate nitrogen‐fixing and non‐nitrogen‐fixing species, respectively; the outer circle indicates the different orders, using the same color scheme as in (A). Black stars represent proteins from the genus *Glycine*, with red stars highlighting *GmNod19‐19* and *GmNod19‐23*. (C) Single‐cell‐type expression of *GmNod19* family genes *Gm05G126800*, *Gm08G081700* and *Gm09G025200*. (D) Heatmap representation of the tissue‐specific expression patterns of the *Nod19* gene family in soybean. Data were obtained from SoyBase (https://www.soybase.org) and are shown as log_2_‐normalized TPM values. (E) Structural characterization of *Nod19* genes in representative species. (F) Illustration of localized gene duplications resulting in *Nod19* gene clusters along soybean chromosomes. The number of exons for each gene is given above their diagram. (G) Multiple DNA sequence alignment of the promoter regions of representative *GmNod19* genes. Due to space limitations, only regions near the transcription start site (TSS) are shown. (H) Distribution of WRKY transcription‐factor binding sites (W‐box motifs, 5'‐TTGAC[CT]‐3') identified in the 2‐kb upstream regulatory sequences of eight genes belonging to the soybean *Nod19* superfamily.

The Stress up‐regulated Nod19 (SURNod19) protein domain is one of the main structural domains in proteins that are either specific to soybean or unique to the legume family [10]. Here, certain transcripts bearing sequences encoding this domain (i.e. *Nod19‐19* and *Nod19‐23*) are differentially expressed in a cell‐type‐specific manner in response to rhizobial infection and are induced by select WRKY transcription factors (Figure 4). To investigate whether the stress up‐regulated Nod19 family is associated with legume‐specific adaptation and evolution of nodulation, we identified 307 proteins containing the SURNod19 domain from 35 species (Tables S10 and S11), comprising 27 species from four nitrogen‐fixing orders (Cucurbitales, Fagales, Rosales, and Fabales), one species from Vitales, and seven species from Poales, Lamiales, Solanales, and Malphigiales used as outgroups. The dataset used here is the same as that used by Griesmann et al. (2018), with the addition of five perennial wild soybean (*Glycine soja*) accessions [42]. This protein family underwent expansion in the Fabales. By contrast, the number of identified genes in the other three nitrogen‐fixing clades does not differ from that in non‐nitrogen‐fixing clades, with most species having only one or two copies (Figure 7A). In the Cucurbitales, Fagales, and Rosales, we detected no gene amplification for the *Nod19* family regardless of nitrogen‐fixation status. However, within the Fabales, we found much more genes encoding proteins with a Nod19 domain in non‐nitrogen‐fixing species, similar to the number observed in the genome of nitrogen‐fixing *L. japonicus* (Figure 7A). In addition, the *Nod19* gene family also underwent some expansion in the non‐nitrogen‐fixing species common flax (*Linum usitatissimum*), although not to the same extent as in Fabales (Figure 7A). Based on these observations, we propose that the expansion of the *Nod19* gene family in a given species is unlikely to have been a decisive factor related to nitrogen‐fixing ability.

Phylogenetically, based on the conserved SURNod19 domain, most Nod19 proteins cluster in distinct species‐specific clades (Figure 7B), indicating that expansion of this family occurred mainly in a species‐specific manner. Members of two small clades containing GmNod19‐19 and GmNod19‐23, respectively, likely originated from the common ancestral genes before the divergence of *Glycine* and common bean (*Phaseolus vulgaris*). Moreover, genes belonging to these two clades have not undergone large‐scale gene amplification in different species, indicating that their functions are relatively evolutionarily conserved. Of Nod19s within each species‐specific clade, most tightly related proteins are encoded by genes physically located close to each other (Table S11), suggesting that the expansion of the *Nod19* gene family may have arisen mainly from localized gene duplication.

To understand the relevance of the *Nod19* family expansion on soybean nitrogen‐fixation ability, a comparative analysis of protein sequences and tissue‐level expression patterns was carried out (Figure 7C–F). The soybean genome encodes 24 predicted Nod19 proteins that share conservation of the SURNod19 domain. Within these, we identified three to five conserved motifs; apart from motif 2 being absent in the protein encoded by *Gm09G025200* and motif 3 being lost in the protein encoded by *Gm03G057700*, all other motifs were shared among soybean Nod19 proteins, showing no major sequence differences (Figure S19). In terms of *GmNod19* gene structures, mainly two types of structures are found. One consists of genes in the same clade as *Nod19‐19* and *Nod19‐23*, which all contain three exons. The remaining genes mostly have four exons, except for one gene on chromosome 1 that is annotated with seven exons (Figure 7E, F). A comparison of the two gene structures with their homologs in other outgroup species suggested that the three‐exon structure likely represents the older copy.

In our scRNA‐seq data, besides the DEGs *GmNod19‐19* and *GmNod19‐23* identified in response to rhizobial infection, we detected expression of only three other *Nod19* superfamily genes: *Gm05G126800*, *Gm08G081700*, and *Gm09G025200* (Figure 7C). All three belong to the same clade as *GmNod19‐19* and *GmNod19‐23*. Among these five genes, *Gm05G126800*, *Gm08G081700*, and *GmNod19‐19* are more similar in sequence, whereas *Gm09G025200* is more closely related to *GmNod19‐23* (Figure 7D). Although *Gm05G126800* and *Gm09G025200* were expressed, their expression levels were quite low. *Gm05G126800* was primarily expressed in procambium cells, but *Gm09G025200* was expressed only in a few epidermal cells. *Gm08G081700*, despite having a relatively high expression level, did not exhibit cell‐type‐specific expression and did not respond to rhizobial infection. In transcriptome data in different soybean tissues obtained from SoyBase, except for *Gm15G131300*, other expanded *Nod19* genes did not group in the clade containing *GmNod19‐19* and *GmNod19‐23* and appear to not be expressed in our scRNA‐seq root datasets or in other tissues. Thus, these amplified genes are unlikely have an obvious effect on nodulation ability.

Local duplications appear to be the primary means driving the expansion of the *Nod19* gene family (Figures 7E, F; Figure S20), resulting in a series of genes with highly similar sequences. Apart from non‐expressed genes, our scRNA‐seq data suggested that the expression patterns of the remaining genes are not identical, despite sharing highly similar sequences, as for example *Gm05G126900* and *GmNod19‐19*, located on chromosome 5, and *Gm15G131300* and *GmNod19‐23*, on chromosome 15. Examination of their local arrangement on the chromosome revealed that *Gm05G126800* and *GmNod19‐19* represent two inverted duplications inserted into another *Nod19* gene, which was split into three parts that were named *Gm05G126700*, *Gm05G126750*, and *Gm05G127100* in the v4 version of the Williams 82 reference genome, although these represent three fragments of a complete *Nod* gene (Figure 7F; Figure S21). Downstream of *Gm08G081700*, we also detected another apparently degraded *Nod19* gene, suggestive of a potential exchange between the two segments. On chromosome 4, *Nod19* genes initially underwent several tandem duplications, followed by an inversion duplication within the duplicated region. Compared to the gene pairs on chromosomes 5 and 15, the tandem repeats on chromosome 4 are spaced farther apart, separated by transposon sequences (Figure 7F). Additionally, we identified a long terminal repeat (LTR)‐type transposon in the inverted duplicated segment on chromosome 3, suggesting that the amplification on this chromosome was primarily mediated by transposons. We saw a similar situation for chromosomes 1 and 3, where LTR‐type transposons were present within the expanded *Nod19* gene regions. This pattern may help explain why these genes were not expressed following the duplication, which is presumably due to silencing imposed by the nearby transposons.

To investigate the differential expression patterns of *GmNod19* genes observed in various tissues and cells, we analyzed their promoter regions (Figures 7G; Figure S22). Surprisingly, except for *Gm15G131400* and *GmNod19‐23*, which have a high degree of sequence similarity in a small segment very close to the transcription start site, promoter regions of the other genes bear substantial differences (Figures 7G). Most of promoter regions were rich in AT‐rich simple repeat sequences, and some also contained multiple tandem repeats (Figure S22). In the 2‐kb upstream regulatory region of all soybean *Nod19* family genes, WRKY transcription factor binding sites (5'‐TTGAC[CT]‐3') were identified only in *Nod19‐19* and *Nod19‐23*, as well as their homologous genes within the same subclade (Figure 7D), excluding the duplicated genes on chromosome 3 (Figure 7H). Two consecutive WRKY binding sites are present in the *GmNod19‐23* promoter. These findings suggest that the observed sequence amplification and recombination within *Nod19* gene family may also result from the tandem repeats of promoter regions and further lead to the differentiation of the promoters, explaining why they confer different tissue‐ and cell‐level expression patterns. We infer that the functional differentiation of the *Nod19* gene family members during nodulation is likely to have been associated with the diversification of their promoter regions.

## 3 Discussion

Tremendous efforts have been made to identify determinant genes of the rhizobium–legume symbiosis and to construct the underlying GRN through transcriptome profiling. Here, using scRNA‐seq, we sampled mock‐treated soybean roots and inoculated roots at 4 dpi with *B. japonicum* for transcriptome profiling to identify genes and pathways involved in the early stages of symbiosis, particularly genes with roles in the formation of the infection threads and nodule primordia. We were particularly interested in transcriptomes of epidermal cells and cortical cells, because the perception of Nod factors, root‐hair curling, infection‐thread formation, and progression through the cortex all take place in epidermal cells, but the nodule infrastructure is formed in cortical cells [24]. We confirmed that epidermal and cortical cells express the largest number of DEGs between mock‐ and *B. japonicum*‐inoculated roots during the early stages of rhizobial infection. The cell‐specific expression data presented here, particularly the DEGs identified in epidermal and cortical cells, serve as a starting point for generating hypotheses for identification of SNF‐related genes and for the dissection of the underlying genetic reprogramming during the formation of infection threads and nodule primordia.

Successful nodulation requires crosstalk between Nod‐factor signaling and innate immunity to overcome the plant defense response, as well as providing spatiotemporal coordination of epidermal and cortical gene‐expression programs [43]. The GO‐term enrichment analysis of the DEGs and the GRN centered around transcription‐factor genes constructed here indicated how hormones, particularly ethylene, mediating cell‐type‐specific immune responses and intercellular communication, are crucial factors ensuring successful rhizobium infection and spatially and temporally proper formation of nodules. Within the reconstructed GRN, genes associated with ethylene and auxin signaling are organized into two distinct and largely independent regulatory modules (Figure S10). Ethylene predominantly coordinates with JA and SA, suggesting a role in fine‐tuning defense and immune homeostasis during infection. In contrast, auxin associates primarily with JA and ABA, indicating its involvement in integrating developmental reprogramming with stress responses. In general, ethylene, SA, gibberellins, brassinosteroids, and cytokinin are considered negative regulators, whereas auxin promotes epidermal infection in legumes [24]. The role of ethylene in soybean nodulation remains controversial; although ethylene production has been reported to be enhanced in soybean roots after inoculation with *B. japonicum* [44], independent studies have shown that increased ethylene production has no effect on nodule formation in soybean [45], whereas the ethylene response factors *GmENS1* and *GmENS2* were reported to promote nodule senescence [46]. The differential effects of ethylene on root nodulation are proposed to be cultivar‐dependent, potentially reflecting genetic selection during breeding processes [47, 48] or to arise from differences in experimental methodologies [45]. Our phenotypic analyses of nodule numbers following ACC and AVG treatments in different cultivars further support this view. Moreover, even in Williams 82, we found that knockdown of ethylene biosynthesis genes (*ACS*) had a much less pronounced effect on nodule number compared to the individual knockdown of downstream ethylene‐responsive genes *GmWRKY6.3/6.4* and *GmNod19‐19/23* (Figure 5B; Figure 6S), indicating the pleiotropic nature of ethylene and the complexity of its regulatory role in soybean nodulation.

In Williams 82, our data identified ethylene as an upstream positive regulator of a now‐defined infection‐promoting regulatory circuit involving *GmWRKY6.3/6.4* and their downstream targets *GmNod19‐19/23*, and link ethylene biosynthesis to nodulation outcomes in soybean. Specifically, down‐regulation of ethylene biosynthesis by targeting *GmACS1* and *GmACS2* by RNAi lowered endogenous ethylene levels and reduced gene expression, supporting a causal connection between ethylene availability and activation of this regulatory circuit. Perturbation of candidate genetic components produced strong and consistent nodulation phenotypes and selectively impaired infection‐thread‐associated outputs, indicating that the pathway comprising *GmWRKY6.3/6.4* and targets *GmNod19‐19/23* operates primarily during the early infection phase to promote infection‐thread formation (Figure 5G–J). Therefore, ethylene can exert a net positive effect at early stages by activating a transcriptional program that facilitates infection progression, even if it may have neutral or negative effects on other nodulation stages or tissues in soybean.

Our single‐cell transcriptome further supports a spatially resolved mode of ethylene regulation. In these data, two *ERF* genes were up‐regulated in epidermal and cortex cells, whereas most *ERF* genes were down‐regulated in infected cells, suggesting that cell‐type‐specific regulation of the ethylene pathway is crucial for successful infection. Such differential *ERF* responses across cell types provide a plausible mechanism by which ethylene signaling can be tuned to support permissive infection in outer tissues while avoiding excessive ethylene responses after rhizobial entry, thereby enabling efficient infection‐thread progression. Altering ethylene biosynthesis is expected to affect multiple developmental and defense processes in parallel, and the final nodulation output likely reflects the net balance among competing ethylene‐dependent pathways. By contrast, the *GmWRKY6.3/6.4*‐dependent pathway represents a more proximal regulatory bottleneck for infection‐thread progression, so manipulating these genes apparently results in stronger, stage‐focused effects. Together, our results support a model in which ethylene contributes positively to soybean nodulation by activating an early‐infection pathway in a cell‐type‐dependent manner, while the overall ‘ethylene phenotype’ reflects integration of multiple ethylene‐responsive programs across tissues and stages.

Given the potential importance of *GmNod19‐23*, as well as *GmNod19‐19*, in early root‐nodule infection, we conducted an evolutionary analysis of the entire *Nod19* gene family. Our analysis of gene expansion and expression of *Nod19* members in various tissues before and after rhizobium infection revealed that family expansion appears to have taken place mainly through transposon‐mediated mechanisms of lineage‐specific expansions, occurring independently in various representative species within the Fabales we interrogated. In soybean, except for the subfamily containing *GmNod19‐19* and *GmNod19‐23*, other transposon‐mediated copies appear to be dormant in the genome (i.e., not transcribed). The clade containing *GmNod19‐19* and *GmNod19‐23* likely represents the ancestral copy shared across multiple species, nodulating and non‐nodulating alike. Further analysis of their cell‐specific expression patterns indicates that even *Nod19* genes within the same subfamily have substantially different expression pattern. The functional diversification of the *Nod19* family may primarily arise from the differentiation of their regulatory sequences; what drives the high differentiation of their regulatory regions is unclear, although we wonder if it might be related to transposition or simple repeat sequences present within their promoter regions. Lineage‐specific expansions play an important role in the expansion and differentiation of proteomes of multicellular eukaryotes. Investigation of LSEs in five eukaryotic species revealed proteins involved in the recognition and binding of pathogens and xenobiotics and in environmental stress as being a major target of LSEs [49]. Although most of the expanded *Nod19* genes in soybean appear not to be expressed, these genes may serve as template for generating genetic diversity required to counter rapidly changing pathogens and to respond to other environmental factors. The observed transposon‐mediated lineage‐specific expansion of the *Nod19* gene family and the subsequent differentiation of their regulatory sequences may have influenced the differentiation between nitrogen‐fixing and non‐nitrogen‐fixing species to a certain extent.

A limitation of this study is that each sample included only two technical replicates and no biological replicates. Therefore, our analyses primarily capture technical consistency rather than biological variability across individuals or growth conditions. This design may reduce statistical power and may affect the generalizability of differential expression signals. Although the major trends were reproducible across technical replicates, the conclusions should be interpreted with caution and further validated using independent biological replicates in future studies.

Another potential limitation of our study is that protoplast isolation step itself may induce stress‐related transcriptional changes, which could introduce artifacts or mask subtle biological differences [50]. Although both R and N group samples were processed in parallel using identical protocols to minimize technical bias, this caveat should be considered when interpreting these single‐cell transcriptomic results, especially when stress‐responsive genes are of interest and found to be induced.

Collectively, our data show the feasibility of using scRNA‐seq for constructing cell‐type‐specific GRNs and identifying pathways and genes that contribute to establishing symbiosis during early rhizobium infection in soybean roots. The transcription factors and DEGs located within the GRN are ideal candidates for future hypothesis‐driven functional studies to advance our knowledge of symbiotic dialog.

## 4 Experimental Section

### 4.1 Plant Materials and Growth Conditions

The soybean (*Glycine max* L. Merrill) cultivar Williams 82 was used for generation of single‐cell RNA‐seq libraries and for functional characterization of select candidate genes. Plants were grown in a growth chamber under a 16‐h light/8‐h dark photoperiod (28°C/20°C [day/night], 50% relative humidity) with LEDs lights at an intensity of 800 µmol photons m^−2^ s^−1^. Vermiculite was added to commercial soil (PINDSTRUP) in a 1:1 (w/w) ratio.

Nicotiana Benthamian was grown under 28°C/22°C [day/night], 70% relative humidity, with LEDs lights at an intensity of 150 µmol photons m^−2^ s^−1^. Plants at 5–6 weeks of age were used for Agrobacterium‐mediated transient transformation.

### 4.2 Protoplast Isolation and Single‐Cell RNA‐seq Library Construction

For protoplast isolation, procedures were used as described by Jia et al. [51] with some modifications. Roots of 8‐d‐old seedlings at the early vegetative (V2) growth stage were harvested from at least 10 seedlings 4 d after mock‐inoculation with distilled water or inoculation with *Bradyrhizobium japonicum* USDA110 at OD_600 nm_ = 1. For both the inoculated and control groups, we collected entire lateral roots (from base to tip) rather than root segments. In the inoculated group, only lateral roots displaying a visible early nodule bump at 4 days post‐inoculation (dpi) were selected. For the control group, lateral roots were harvested from corresponding positions with comparable growth status. For each condition, samples were pooled from six plants, with two lateral roots collected per plant. Root fresh weight was not used as a sampling criterion; instead, sampling was standardized by root type, position, and developmental status. Roots were washed using tap water to remove soil debris, then submerged in plasmolysis buffer containing 0.8 m mannitol dissolved in distilled water in a Petri dish, and cut into 0.5–1‐mm segments with a surgical blade. After pre‐washing, samples were individually weighed, transferred to 20 mL of digestion buffer in a clean Petri dish, and subjected to a 30‐min vacuum infiltration at 0.7 kg/cm^2^ to accelerate cell‐wall digestion. The dishes were incubated at room temperature for 4 h with gentle shaking (40 rpm) in the dark. After digestion, the protoplast suspension was placed on ice, filtered through one 70‐µm cell strainer and two 40‐µm cell strainers, and then transferred to 50‐mL conical tubes. Protoplasts were collected by centrifugation at 400 rcf for 2 min at 4°C before being washed with 10 mL pre‐cooled W5 buffer, collected again by centrifugation under the same conditions as above, and finally resuspended in 300 µL plasmolysis buffer. Cell number was determined using a hemocytometer and cell density was adjusted to ∼1,000 cells/µL. About 10,000 cells for each replicate were loaded onto a 10X Genomics Chip A with V3 chemistry, aiming to capture 6,000 cells per sample. Sequencing was performed by Novogene Ltd. Co. In total, four samples (duplicates for each of the two treatments) were sequenced.

### 4.3 scRNA‐seq Data Pre‐Processing

Quality control, sample de‐multiplexing, and barcode processing were performed with the Cell Ranger Single Cell Software Suite (v2.1.1) of 10X Genomics (http://10xgenomics.com/). The reference genome of Williams 82 Assembly 4 (Wm82.a4) [10] was retrieved from SoyBase (www.soybase.org), and gene indices were generated using the Cell Ranger Single built‐in command “*mkref”*. The de‐multiplexed single‐cell reads in FASTQ format from each sample were aligned to the reference genome independently with the Cell Ranger count pipeline and converted to digital gene‐expression matrices (cellranger count). Between‐sample normalization and data integration were performed using the Cell Ranger aggr pipeline, to concatenate the transcript count tables (cellranger aggr –normalize = mapped). Cells with fewer than 200 detected genes and genes expressed in fewer than three cells were removed. The total number of genes identified for each cell together with unique molecular identifier counts were examined to identify outliers.

### 4.4 Cell Clustering and Dimensionality Reduction

The aggregated filtered feature matrix files generated by Cell Ranger were loaded into scanpy platform v1.10.4 [52] to filter and normalize data and perform subsequent analyses. Cells expressing fewer than 200 genes or more than 3,000 genes were removed using the “sc.pp.filter_cells” function and genes expressed in fewer than three cells were also removed with the “sc.pp.filter_genes” function. The resulting data were then normalized and standardized using the “sc.pp.normalize_total” function (target_sum = 1e4) and the “sc.pp.log1p” function, respectively. Principal components were calculated using only genes with highly variable expression across cells, selected by the “highly_variable_genes” function. The neighborhood graph was calculated with the first 30 corrected principal components (n_neighbors = 10, n_pcs = 30) and then partitioned into clusters using the Leiden algorithm (resolution = 0.5, flavor = “igraph”, n_iterations = 2) and visualized with the uniform manifold and approximation projection (UMAP) dimensionality reduction method.

### 4.5 Cell‐Type Annotation

Marker genes for each cluster were computed using the Wilcoxon rank‐sum test in Scanpy [52] with overestimated variance. The identified markers were then used for searching known markers in soybean roots from the literature [20, 21, 22]; the expression of each identified marker gene among all clusters was visualized for cell‐type assignment. For clusters lacking high‐specificity markers, previously identified root cell types from *Arabidopsis thaliana* [19] and *Medicago truncatula* [27, 28] genes were used. For each cluster, the top‐20 differentially expressed genes (DEGs) (*p* < 0.01) were ranked and compared to all other clusters, after which these DEGs were used to assign cell‐type identity to each cluster manually. For cell‐type classification in infected cells, we integrated results from two cell‐identity assignment pipelines: Scanpy [52] and decoupler [53]. Cells from the N dataset were firstly clustered using the same parameters described above. Infected cells were extracted from the annotated AnnData object and subjected to cell‐type classification using both Scanpy and Decoupler. A cross‐tabulation table was generated to summarize the agreement between the two programs assignments for three categories: cortex_like, epidermis_like, and mixed. Cells classified identically by both methods were assigned their consensus label, while cells with discordant assignments were categorized as mixed.

### 4.6 Extraction of Infection‐Cell‐Specific Genes

Genes specifically expressed in infected cells were identified from the infected‐group dataset using the Scanpy [52] analysis framework. For each gene, the average expression level and the fraction of expressing cells were calculated separately for infected cells and all other cell types. Infection‐cell‐specific genes were defined using the following criteria: expression fraction ≥ 30% in infected cells, expression fraction ≤ 5% in other cell types, mean expression ≥ 0.5 in the infected group, fold change ≥ 10, and *p < 0.05*.

### 4.7 Gene Set Variation and Gene‐Ontology Enrichment Analysis

Identification of DEGs between the mock‐inoculated and inoculated samples was performed separately for each cell type using the pyDEG method [54] in omicverse (*p* < 0.05). The identified DEGs in each cell cluster were further examined and subjected to gene‐ontology (GO) term enrichment analysis using the enricher function in clusterProfiler [55]. Enrichment results were further reduced using the simplify function with a cutoff of 0.6 to remove redundant terms, and only biologically relevant pathway categories were retained for visualization. The GO annotation file of Wm82.a4.v1 genome was retrieved from SoyBase and used for assigning GO terms to each gene.

### 4.8 Cell‐Trajectory Analysis and Transcription‐Factor Gene‐Regulatory‐Network Construction

For cell trajectory inference, previously calculated PCA space and neighborhood graphs were used to draw a single‐cell graph using the “scanpy.tl.draw_graph” function in scanpy. The resulting graph was first denoised by computing diffusion maps using the “scanpy.tl.diffmap” function with the first‐ten principal components. Neighborhood distances were calculated between diffusion components, and the graph was drawn on the diffusion map using the “scanpy.pp.neighbors” and the “scanpy.tl.draw_graph” commands, respectively. Previously calculated UMAP clusters were used to generate a partition‐based graph abstraction (PAGA) graph using the “scanpy.tl.paga” command. Finally, the single‐cell graph was recomputed using the “scanpy.pl.draw_graph” command based on the PAGA graph. Differentially expressed transcription‐factor genes were identified and classified based on PlantTFDB [56] (https://planttfdb.gao‐lab.org/). The gene regulatory network (GRN) was constructed using RegDiffusion [23] (https://github.com/TuftsBCB/RegDiffusion). The RegDiffusion Model was trained for 1,000 iterations using the provided trainer with the command “rd_trainer.train” and the GRN was constructed using the “rd_trainer.get_grn” function.

### 4.9 In situ Hybridization

Lateral root and nodule primordia at 4 dpi were fixed in 4% (w/v) paraformaldehyde/PBS (pH 7.2) at 4°C overnight after excision, dehydrated through an ascending ethanol series, cleared in xylene, and embedded in paraffin at 60°C. Serial sections (8‐µm) were cut, mounted on poly‐L‐lysine‐coated slides, and baked at 42°C for 2 h. A cDNA fragment of the transcript of interest was cloned into the pEASY‐Blunt3 vector (Trans, CB301) and used as template for in vitro transcription with SP6/T7 RNA polymerases (Roche) in the presence of digoxigenin‐UTP (Roche) to generate antisense and sense RNA probes. Probes were fragmented to an average length of 150 nt by alkaline hydrolysis (0.2 M NaHCO_3_/0.2 mM Na_2_CO_3_). Sections were deparaffinized in xylene, rehydrated through a descending ethanol series, permeabilized with proteinase K (1 µg mL^−1^, 37°C for 27 min, Roche), and post‐fixed in 4% w/v paraformaldehyde for 10 min. Hybridization was performed overnight at 42°C with 200 ng mL^−1^ probe in 50% v/v formamide‐containing hybridization buffer. After hybridization, slides were washed four times in 0.2× SSC at 55°C (15 min each), blocked twice with 1% w/v BSA (45 min each), and incubated with alkaline phosphatase‐conjugated anti‐digoxigenin antibody (1:1,000, Roche) for 3 h at room temperature. Signals were developed using NBT/BCIP substrate (Roche) under light‐shielded conditions until optimal intensity was reached. Images were captured with an Olympus BX51 microscope.

### 4.10 Vector Construction for Over‐Expression

For the construction of over‐expression plasmids, primers were designed using Primer3 and their sequences were compared to the reference genome by BLAST [57] to ensure their specificity. The full‐length coding sequence of target genes was amplified from Williams 82 root cDNA and the resulting PCR products were ligated into the 35S‐pBI121 vector [58], transformed into *Escherichia coli* DH5α, and confirmed by Sanger sequencing.

### 4.11 Vector Construction for RNAi

For generation of the RNAi plasmids, a DNA fragment 200–400‐bp long was amplified from coding sequences of interest based on regions with high similarity and gene specificity. The specificity of the selected sequence was verified by conducting a whole‐genome BLAST search [57] to ensure that only the target genes would be silenced. Two pairs of homologous recombination primers for the pG2RNAi2 vector were designed using SnapGene based on the sequence‐verified fragment. The first pair of homologous recombination primers contained *Asc*I and *Swa*I restriction sites, while the second pair contained *Avr*II and *Bam*HI sites, ensuring that the fragment was directionally inserted in an inverted orientation rather than reverse complementary after two consecutive cloning steps. Primers were synthesized by Sangon Biotech (Shanghai). PCR amplification was performed using Phanta Max Super‐Fidelity DNA Polymerase (Nanjing Vazyme Biotech) to generate two fragments. The pG2RNAi2 vector was digested with fast‐cutting endonucleases (New England Biolabs) at the *Asc*I and *Swa*I sites, followed by homologous‐recombination‐mediated ligation using ClonExpress Ultra One Step Cloning Kit V3 (Nanjing Vazyme Biotech). The ligation product was then transformed into *E. coli* DH5α. Positive clones were identified by sequencing at Sangon Biotech (Shanghai). After successful verification of the first round of cloning, a second round of ligation was performed using the same protocol with the *Avr*II and *Bam*HI sites. The RNAi construct was confirmed by sequencing and subsequently used for plant transformation.

### 4.12 Soybean Hairy‐Root Transformation

Over‐expression and RNAi plasmids developed as described above were individually transformed into *Agrobacterium rhizogenes* K599 and colonies harboring the construct of interest were first spread onto solid YEP medium, incubated for 2 d until colonies formed, and then cultured in liquid YEP medium at 28°C for 10 h. The cultivar Williams 82 was used as recipient material for *A. rhizogenes*‐mediated transformation. Seeds were sown on soil (vermiculite and nutritional soil mixed in a 1:1 [w/w] ratio) and allowed to grow for 6 d before four wounds were applied below the cotyledons using a 1‐mL syringe and smeared with *A. rhizogenes* cell suspensions harboring either the empty vector (EV), the over‐expression vector (OE), or the RNAi vector. Seedlings were then covered with a plastic bottle to maintain high humidity for 6 d. When hairy roots grew to 5–10 cm in length, the taproot was cut off and cultured for another 3 d before inoculation with *B. japonicum*. Phenotypic data were collected at 28 dpi. At least 10 seedlings for each treatment and plasmid were used and the whole experiment was performed three times. The expression level of the target gene was examined in each individual hairy root, and only those OE or RNAi roots showing the expected upregulation or knockdown were included for subsequent phenotypic quantification.

### 4.13 ChIP–qPCR

Roots harvested from hairy‐root lines over‐expressing GmWRKY6.3 and GmWRKY6.4 with a GFP tag at the C‐termini at 4 dpi were collected in pre‐chilled 1× PBS buffer on ice. Chromatin was cross‐linked by incubating the tissue in 1% v/v formaldehyde in 1× PBS under vacuum (7.5 psi) for 3 min, repeated three times. Cross‐linking was quenched by adding 0.125 m glycine in 1× PBS under the same vacuum conditions. After washing with 1× PBS, 1 g of cross‐linked tissue was ground in liquid nitrogen and resuspended in 20 mL pre‐chilled ChIP Extraction Buffer I, then centrifuged at 4,000 rpm for 20 min at 4°C. The pellet was sequentially washed with ChIP Extraction Buffer II (12,000 rpm, 10 min) for 1–4 cycles depending on chlorophyll content, and then incubated in 300 µL ChIP Extraction Buffer III. After centrifugation at 13,000 rpm for 1 h at 4°C, the nuclear pellet was resuspended in 100 µL Nuclear Lysis Buffer and incubated on ice for 30 min. Chromatin was sonicated after adding 200 µL ChIP Dilution Buffer, and the supernatant was collected after centrifugation (13,000 rpm, 10 min at 4°C). A 50‐µL aliquot was reserved as input control. The remaining sample was incubated with 7 µg of antibody pre‐bound to protein A/G Dynal beads overnight at 4°C with rotation. After sequential washes with Low Salt Washing Buffer, High Salt Washing Buffer, LiCl Washing Buffer, and TE Buffer (10 min each at 4°C), DNA was eluted with 200 µL ChIP Elution Buffer at 65°C. Cross‐linking was reversed by adding 5 M NaCl and incubating at 65°C for at least 8 h. DNA was purified following treatment with proteinase K (1 µL of 20 mg/mL) and RNase A (20 µg) by phenol–chloroform extraction and ethanol precipitation. Real‐time quantitative PCR was performed using 2× SYBR qPCR Master Mix with gene‐specific primers. Diluted ChIP and input samples were added to a 96‐well plate with the primer mix, sealed, and centrifuged before being placed in a real‐time qPCR instrument (Applied Biosystems, USA). The PCR program consisted of an initial denaturation step at 95°C for 30 s (1 cycle), followed by 40 cycles of amplification (95°C for 10 s, 60°C for 30 s), and a final melt‐curve analysis. Statistical analysis was conducted using Student's t‐test and significance was defined as *p* < 0.05.

### 4.14 Yeast Two‐Hybrid Assays

Yeast two‐hybrid (Y2H) assays were performed using the Matchmaker Gold Yeast Two‐Hybrid System (Clontech, Shanghai, China), following the procedure described by Wang et al. [59]. Full‐length coding sequences of *GmWRKY6*.3 and *GmWRKY6*.4 were cloned into pGADT7 and pGBKT7, respectively, to generate the recombinant constructs encoding WRKY6.3–AD and WRKY6.4–BD. Transformed yeast strains were plated on double‐dropout medium (DDO/X‐alpha–Gal; ‐Leu/‐Trp) and quadruple‐dropout medium (QDO/X‐alpha–Gal; –Ade/–Leu/–Trp/–His), and protein‐protein interactions were assessed based on yeast growth and reporter activation. PCR primers used for vector construction are listed in Supplementary Table S12.

### 4.15 Bimolecular Fluorescence Complementation assays

For Bimolecular Fluorescence Complementation (BiFC) assays in *N. benthamiana*, the coding sequences of GmWRKY6.3 and GmWRKY6.4 were cloned into the YNE and YCE vectors to generate GmWRKY6.3–nYFP and GmWRKY6.4–cYFP fusion constructs, respectively, that harbor sequence encoding the N‐ and C‐terminal fragments of YFP. The recombinant plasmids were transformed into *A*. tumefaciens strain GV3101 and co‐infiltrated into the abaxial epidermis of 4‐week‐old *N*. benthamiana leaves, following the method of Chen et al. [60]. Fluorescence and bright‐field images were acquired using a laser scanning confocal microscope (TCS SP8, Leica). YFP signals were excited at 514 nm with laser power set to 3%–4.99%. Detection settings were as follows: hybrid detector (HyD; 525–540 nm, gain 10–96.1) and PMT transmitted‐light channel (PMT trans; gain 305–387.3). PCR primers used for vector construction are listed in Supplementary Table S12.

### 4.16 Luciferase Complementation Imaging Assays

Luciferase complementation imaging (LCI) assays were performed as described by Liu et al. [61]. The full‐length coding sequences of GmWRKY6.3 and GmWRKY6.4 were cloned into the pCAMBIA1300‐nLUC and pCAMBIA1300‐cLUC vectors [60], respectively. The recombinant plasmids were transformed into *A*. tumefaciens strain GV3101 carrying the helper plasmid pSoup‐19 and then co‐infiltrated into *N*. benthamiana leaves. Empty vectors were used as negative controls. After spraying the leaves with luciferase substrate (Vazyme, D‐Luciferin Potassium Salt, DD1210‐01), chemiluminescence signals were captured using a Boluteng in vivo imaging system.

### 4.17 Dual‐Luciferase Reporter Assays

The coding sequences of *GmWRKY6.3* and *GmWRKY6.4* were cloned into pGreenII 62‐SK [62] to generate GmWRKY6.3‐62SK and GmWRKY6.4‐62SK, respectively. The promoter of GmNod19‐19 was inserted into pGreenII 0800‐LUC to generate the reporter construct GmNod19‐19‐LUC. These recombinant plasmids were introduced into *A*. tumefaciens GV3101 carrying the helper plasmid pSoup‐19 and co‐infiltrated into *N. benthamiana* leaves. Empty vector was used as the negative control. At 48 h after infiltration, 3–4 tobacco leaf discs of similar size were collected and placed into 2 mL centrifuge tubes preloaded with 3–4 steel beads. Samples were immediately frozen in liquid nitrogen and ground at 60 Hz for 60 s using a tissue grinder. Subsequently, 100 µL lysis buffer was added, and samples were incubated on ice for 5 min, followed by centrifugation at 12,000 rpm for 1 min. The supernatant was collected, and 50 µL of lysate was transferred to a microplate. Then, 100 µL of firefly luciferase reaction working solution and 100 µL of Renilla luciferase reaction working solution were added sequentially to measure firefly luciferase (LUC) and Renilla luciferase (REN) activities, respectively. The assay was performed using the Vazyme Dual Luciferase Reporter Assay Kit (DL101‐01).

For in vivo imaging, a portion of the infiltrated leaves was sprayed with luciferase substrate (Vazyme, D‐Luciferin Potassium Salt, DD1210‐01), and chemiluminescence signals were captured using a Boluteng in vivo imaging system.

### 4.18 Pharmacological Manipulation of Ethylene Signaling

Seedlings of three soybean cultivars (Willisms 82, Qi Huang 34 and Dong Nong 50) were grown to the two‐trifoliate‐leaf stage and inoculated with 50 mL of *B. japonicum* strain USDA110 suspended in distilled water (OD_600 nm_ = 0.08). At 12 h and 48 h post‐inoculation, seedlings were treated with the ethylene precursor 1‐aminocyclopropane‐1‐carboxylic acid (ACC, 10 µM) or the ethylene biosynthesis inhibitor aminoethoxyvinylglycine (AVG, 5 µM); control plants received an equal volume of distilled water. Root tissues were harvested at 4 days post‐inoculation for RNA extraction and RT–qPCR analysis, as well as for observation of infection foci and infection threads. In addition, 20 plants per treatment were retained for assessment of nodulation phenotypes at 28 dpi.

### 4.19 Development of RNAi Constructs for *ACS* Silencing

For simultaneous down‐regulation of *GmACS1* (*Gm05G211700*) and *GmACS2* (*Gm08G018000*), their coding sequences were first aligned using MUSCLE and regions with high homology within these sequences were selected and used as queries in BLAST searches against the Williams 82 reference genome to ensure specificity. Construction of the RNAi vector and functional validation using soybean hairy‐root system are described above.

### 4.20 Quantification of Ethylene Content in Roots

Ethylene production in empty‐vector controls and *GmACS1*/*2* RNAi hairy roots was determined at 4 dpi. Root tissues were harvested, washed to remove soil, and blotted to remove excess water. Fresh roots (∼1–2 g fresh weight) were placed in a sealed 50‐mL glass vial equipped with a closed gas‐collection system connected via glass and rubber tubing. Moisture in the vial was maintained with distilled water and incubated in darkness at 28°C for 12 h to allow ethylene accumulation. Headspace gas samples (30 mL) were then analyzed using a Shimadzu GC‐2010 gas chromatograph equipped with a flame ionization detector and a stainless‐steel column (2 m × 4 mm). The column temperature was set at 70°C, with injection port and vaporization chamber temperatures at 200°C and 110°C, respectively. Hydrogen, air, and nitrogen flow rates were 40, 400, and 15 mL/min, respectively. Ethylene concentration was calculated against a known ethylene standard using the peak‐height ratio: C*_sample_* = (Peak height*_sample_*/Peak height*_standard_*) x C*_standard_*. The ethylene‐production rate was then determined according to the formula: Rate (µL/g/h) = (C_*_sample_* x V*_vial_*)/(t*_incubation_* x m*_sample_*), where V*_vial_* is the vial volume (µL), t*_incubation_* is 12 h, and m*_sample_* is the fresh weight (g) determined by analytical balance.

### 4.21 Recombinant Protein Expression and Purification

*Escherichia coli* BL21 strains harboring *GmWKRY6.3* and *GmWKRY6.4* pMAL‐c2x expression vectors (Novagen, Beijing, China) were cultured in 5 mL of liquid lysogeny broth (LB) containing appropriate antibiotics at 37°C for 12 h with shaking at 200 rpm. The culture was then transferred to 500 mL of fresh antibiotic‐containing liquid LB medium and grown at 37°C until OD_650 nm_ reached 0.6–0.8. Protein expression was induced by adding isopropyl β‐D‐1‐thiogalactopyranoside (IPTG) to a final concentration of 0.5 mM, and the culture was continued at 37°C with shaking at 200 rpm for 3 h. Cells were harvested by centrifugation at 4,000 rpm for 20 min at 4°C. Harvested cells were resuspended in 20 mL of resuspension buffer and lysed by sonication (5 s on, 5 s off, total 15 min). The lysate was centrifuged at 12,000 rpm for 10 min at 4°C, and the supernatant was transferred to a fresh tube. MBP‐affinity resin (1 mL) was packed into a purification column and equilibrated twice with resuspension buffer. The protein supernatant was loaded onto the column and allowed to flow through slowly. Non‐specific binding proteins were removed by washing with 5‐fold column volumes of resuspension buffer. The target protein was eluted with 2 mL of elution buffer, collecting the eluate in 2‐drop fractions and stored at −80°C for further use.

### 4.22 Electrophoretic‐Mobility Shift Assay (EMSA)

Double‐stranded DNA probes were synthesized by Sangon Biotech. Probes were prepared by annealing equal volumes of complementary single‐stranded oligonucleotides at 95°C for 1 min, followed by gradual cooling to 25°C (decreasing 1°C every 8 s). EMSA reactions were performed using a Beyotime chemiluminescence EMSA kit according to the manufacturer's instructions. Briefly, purified proteins and labeled probes were incubated in EMSA/Gel‐shift binding buffer at room temperature for 10 min to eliminate non‐specific binding before adding the labeled probe. The reaction mixture was loaded onto 6% native polyacrylamide gels and electrophoresed in 0.5× TBE buffer at 120 V until the bromophenol blue dye front reached three‐quarters down the gel. DNA was transferred to nylon membranes at 300 mA for 90 min in 0.5× TBE buffer. After transfer, the membrane was cross‐linked using UV light (5–10 cm distance) for 15 min. The membrane was blocked with blocking buffer at 37–50°C for 15 min, incubated with Streptavidin‐HRP conjugate for 15 min, and washed three times with diluted washing buffer (5 min each). Following equilibration with detection buffer for 5 min, the membrane was exposed to BeyoECL Moon working solution (equal volumes of reagents A and B) for 2–3 min at room temperature before imaging.

### 4.23 RT–qPCR

Total RNA was extracted from hairy roots using Trizol reagent (Thermo Fisher Scientific, USA) after the collection of phenotypic data. RNA quality was checked on a 1.2% (w/v) agarose gel. First‐strand cDNA was synthesized using a HiScript II Supermax synthesis kit (Vazyme, Nanjing, China) and qPCR was performed on a Step One Plus Real‐Time PCR system (Applied Biosystems, USA) using ChamQ Universal SYBR qPCR Master Mix (Vazyme, Nanjing, China). At least 10 biological repeats and three technical repeats were performed for each treatment and construct expressed in hairy roots. Ct values were normalized using *GmActin* (*Gm.09G09270*) as as reference gene and fold change was calculated with the 2^−∆∆Ct^ method [63].

### 4.24 Root‐hair‐curling observation

Root from wild‐type seedlings and those treated with ACC or AVG were harvested at 4 dpi. Seedlings transformed with empty vectors (EV) or *GmNod19* RNAi and *ACS* RNAi vectors were scored and root segments (2 cm) from the junction between root and hypocotyl were harvested at 4 dpi, rinsed in sterile PBS for 2 min to remove vermiculite/Perlite particles, and fixed in ethanol: glacial acetic acid (3:1) for 2 h. After three rinses with distilled water, roots were stained with 0.01% w/v methylene blue for 15 min and rinsed three times [64]. Curled root hairs (showing swollen tips and undulating growth direction) were observed using an Olympus BX53 microscope and counted in a 2‐cm root segment. Data represent mean ± SD from 11 roots per genotype.

### 4.25 Infection‐Thread Observation

Seedlings were inoculated with *GUS*‐tagged *Bradyrhizobium japonicum* USDA110 [65]. Root samples were collected at 4 dpi, fixed with PFA [4% (w/v) paraformaldehyde in 1× PBS, pH 7.4] [66] on ice under vacuum for 10 min, and transferred to X‐Gluc solution [50 mM NaH_2_PO_4_, 50 mM Na_2_HPO_4_, 0.5 mM K_4_(Fe(CN)_6_), 0.5 mM K_3_(Fe(CN)_6_), 1 mg/mL X‐Gluc, 0.1% (v/v) Triton X‐100] for incubation at 37°C in darkness for 2–6 h. After three washes with 75% v/v ethanol, infection threads were observed using an Olympus BX51 microscope and counted in a 2‐cm root segment. Data represent mean ± SD from 11 roots per genotype.

### 4.26 Phylogenetic Analysis of the Stress up‐Regulated Nod19 (SURNod19) Superfamily

To investigate the evolutionary differentiation of the Nod19 protein family among nitrogen‐fixing and non‐nitrogen‐fixing organisms, 35 species were selected (Table S11), including species used by Griesmann et al., with the addition of one annual wild soybean and five perennial wild soybean species [31, 42]. Homologous genes for 355 single‐copy genes reported in the genome of the soybean cultivar Williams 82 (av4) [42] were identified in selected species based on reciprocal BLASTP. A phylogenetic tree using data from the 35 species was constructed using the neighbor‐joining method in RapidNJ [67] with 1,000 bootstrap replicates.

For identification of *Nod19* gene family members in each species, a combination of *de novo* and reference‐based approaches were used. First, the HMM model of SURNod19 was downloaded from Pfam (pfam07712) [68] and used for searching annotated proteins in each species with HMMER [69]. The identified gene models from all species were then combined and used for reference‐based gene annotation using GeMoMa [70]. Protein sequences of resulting genes were then subjected to motif analysis using MEME suite [71], and genes missing more than two conserved motifs were removed from downstream analysis. The phylogenetic tree was also computed using the neighbor‐joining method in RapidNJ with 1,000 bootstrap replicates. Tissue‐specific gene expression data were downloaded from SoyBase, and the distribution of transposons in Williams 82 genome was annotated based on SoyTEdb [72].

### 4.27 Statistical Analysis

For scRNA‐seq, root cells were collected from six plants per treatment, and sequenced with two technique repeats. All other experiments were performed with a minimum of ten biological replicates per condition and at least three independent experimental repeats, unless otherwise specified. Statistical analyses and data visualization were performed using Python (v3.10) and R (v4.3). Continuous variables are presented as the mean standard deviation (SD), while categorical variables are presented as counts or percentage (%). Comparisons between two groups were performed using two‐tailed Student's t‐test. Comparisons among three or more groups were analyzed using one‐way ANOVA followed by Tukey's HSD post‐hoc test; the Kruskal–Wallis test was used when normality assumptions were not met (dual‐luciferase reporter assays). *p* < 0.05 was considered statistically significant.

## Author Contributions

Y.B.Z, Y.G and D.J.Z. designed the experiments, Y.B.Z, Y.G, X.Y.G., L.Q.L. and X.M.L. analyzed the data. C.W, Y.J.S, Q.Q.G., H.X.C, G.B.Z, L.L, Y.G, X.F.Z, B.Y.C, J.F.Z. and X.M.L performed the experiments and data curation. Y.B.Z., G.S.Z., Y.X.L. and X.H. performed the bioinformatics analysis and wrote the manuscript. All authors read and approved the manuscript.

## Conflicts of Interest

The authors declare no conflicts of interest.

## Supporting information




**Supporting File 1**: advs76550‐sup‐0001‐SuppMat.pdf.

**Supporting File 2**: advs76550‐sup‐0002‐Tables S1–S12.xlsx.

**Supporting File 3**: advs76550‐sup‐0003‐Figure and tables captions.docx.

## Data Availability

All raw data generated were submitted to NCBI Sequence Read Archive (SRA) Database, and can be accessed using project ID PRJNA919096.
