# Supplementary material for: Single‐cell Transcriptome Profiling Reveals Gene Regulatory Networks and Key Genes in the Root Epidermis and Cortical Cells Associated with Early Nodulation in Glycine Max
Source: Adv Sci (Weinh). 2026 Jul 11:e76550. Online ahead of print. doi: 10.1002/advs.76550 (PMC13355928; doi:10.1002/advs.76550)
Supplement: Supplementary file 1 — Supporting File 1: advs76550‐sup‐0001‐SuppMat.pdf. [file ADVS-9999-e76550-s002.pdf]

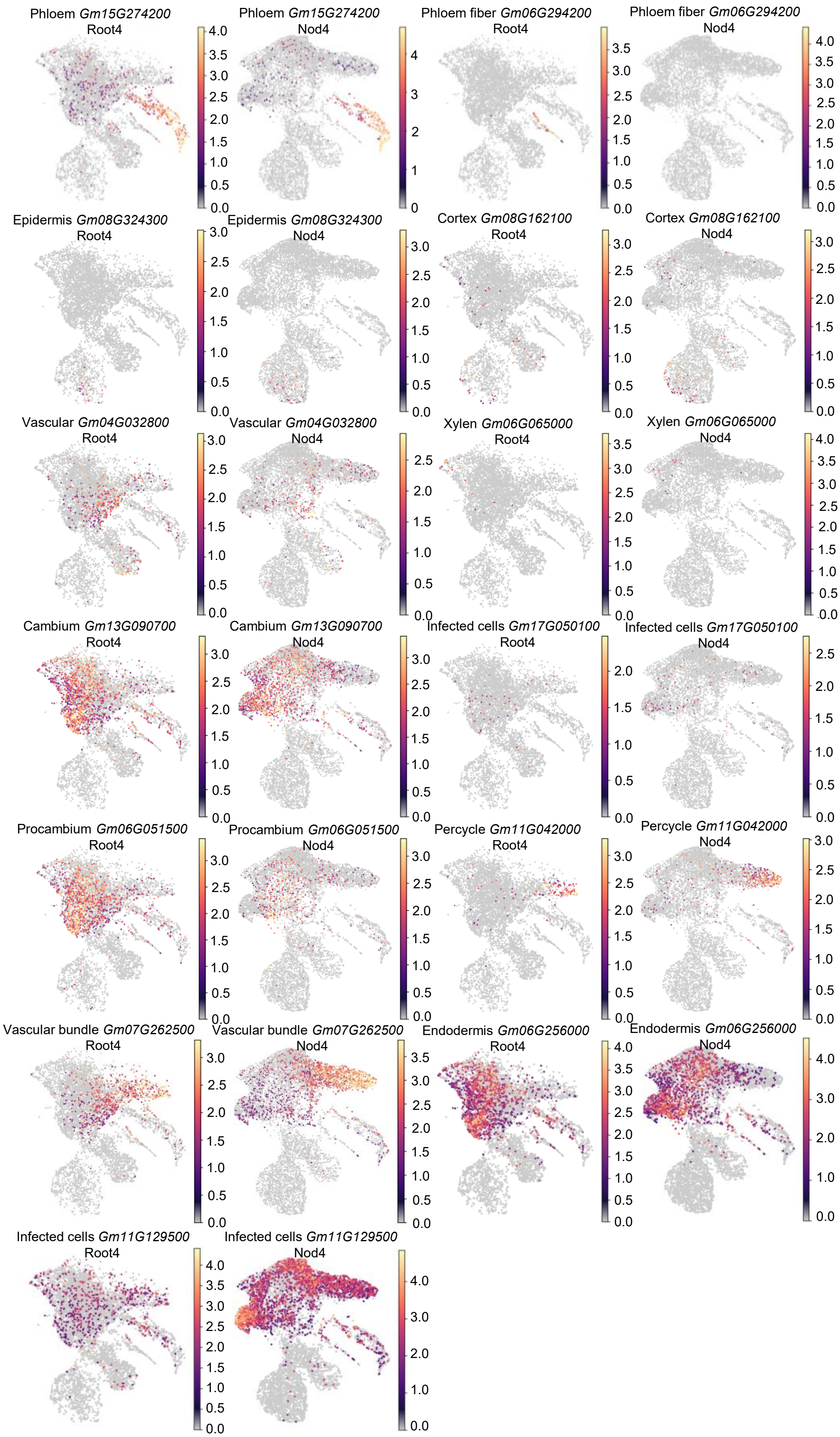
Phloem Gm15G274200 Root4
Phloem Gm15G274200 Nod4
Phloem fiber Gm06G294200 Root4
Phloem fiber Gm06G294200 Nod4
Epidermis Gm08G324300 Root4
Epidermis Gm08G324300 Nod4
Cortex Gm08G162100 Root4
Cortex Gm08G162100 Nod4
Vascular Gm04G032800 Root4
Vascular Gm04G032800 Nod4
Xylen Gm06G065000 Root4
Xylen Gm06G065000 Nod4
Cambium Gm13G090700 Root4
Cambium Gm13G090700 Nod4
Infected cells Gm17G050100 Root4
Infected cells Gm17G050100 Nod4
Procambium Gm06G051500 Root4
Procambium Gm06G051500 Nod4
Percycle Gm11G042000 Root4
Percycle Gm11G042000 Nod4
Vascular bundle Gm07G262500 Root4
Vascular bundle Gm07G262500 Nod4
Endodermis Gm06G256000 Root4
Endodermis Gm06G256000 Nod4
Infected cells Gm11G129500 Root4
Infected cells Gm11G129500 Nod4

Figure S1

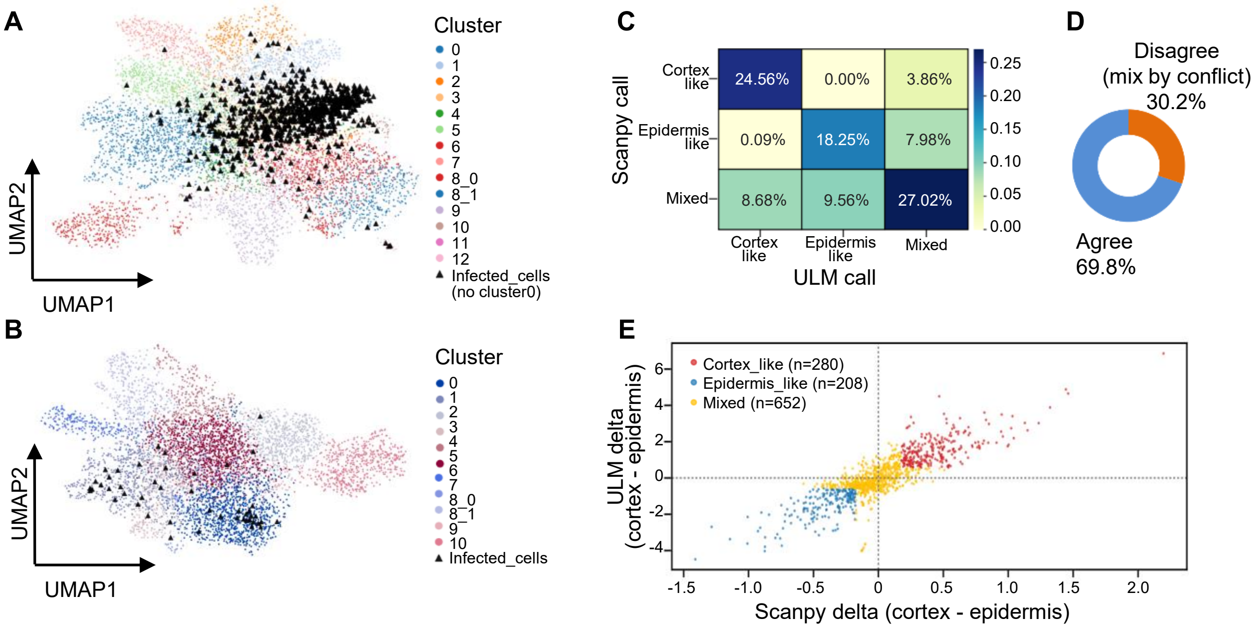
A
Cluster
0
1
2
3
4
5
6
7
8_0
8_1
9
10
11
12
Infected_cells
(no cluster0)
UMAP2
UMAP1
B
Cluster
0
1
2
3
4
5
6
7
8_0
8_1
9
10
Infected_cells
UMAP2
UMAP1
C
Scanpy call
Cortex like
Epidermis like
Mixed
24.56%
0.00%
3.86%
0.09%
18.25%
7.98%
8.68%
9.56%
27.02%
Cortex like
Epidermis like
Mixed
ULM call
0.25
0.20
0.15
0.10
0.05
0.00
D
Disagree
(mix by conflict)
30.2%
Agree
69.8%
E
Cortex_like (n=280)
Epidermis_like (n=208)
Mixed (n=652)
ULM delta
(cortex - epidermis)
Scanpy delta (cortex - epidermis)
6
4
2
0
-2
-4
-1.5
-1.0
-0.5
0
0.5
1.0
1.5
2.0

Figure S2

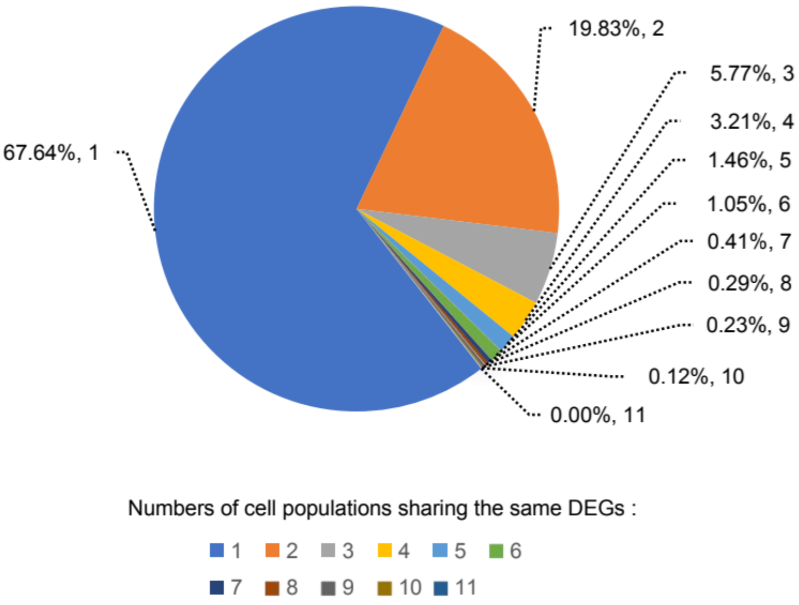
19.83%, 2
5.77%, 3
3.21%, 4
1.46%, 5
1.05%, 6
0.41%, 7
0.29%, 8
0.23%, 9
0.12%, 10
0.00%, 11
67.64%, 1
Numbers of cell populations sharing the same DEGs :
1 2 3 4 5 6
7 8 9 10 11

Figure S3

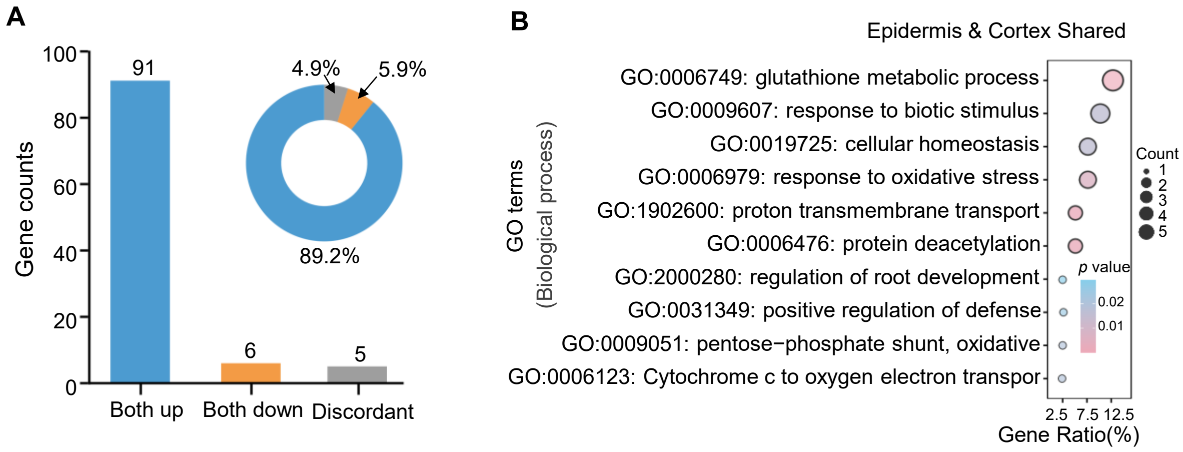
A
100
80
60
40
20
0
Gene counts
91
6
5
4.9%
5.9%
89.2%
Both up
Both down
Discordant
B
Epidermis & Cortex Shared
GO:0006749: glutathione metabolic process
GO:0009607: response to biotic stimulus
GO:0019725: cellular homeostasis
GO:0006979: response to oxidative stress
GO:1902600: proton transmembrane transport
GO:0006476: protein deacetylation
GO:2000280: regulation of root development
GO:0031349: positive regulation of defense
GO:0009051: pentose−phosphate shunt, oxidative
GO:0006123: Cytochrome c to oxygen electron transpor
GO terms
(Biological process)
Count
1
2
3
4
5
p value
0.02
0.01
2.5
7.5
12.5
Gene Ratio(%)

Figure S4

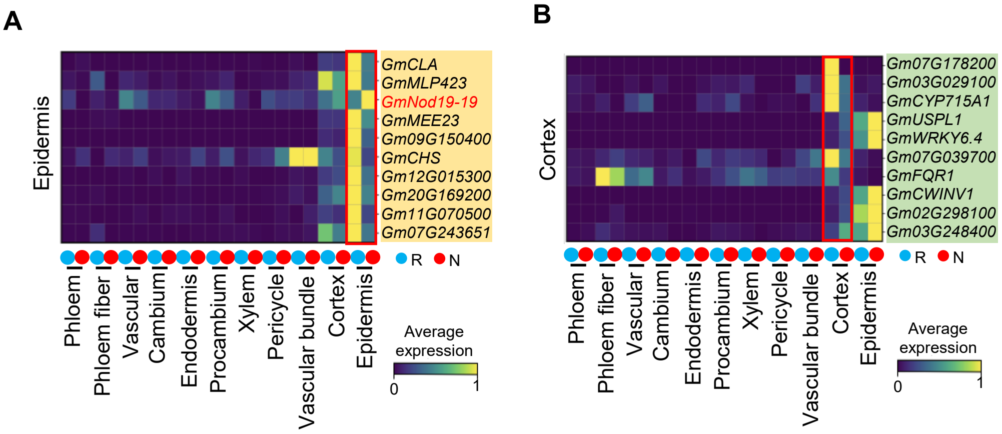
A
Epidermis
GmCLA
GmMLP423
GmNod19-19
GmMEE23
Gm09G150400
GmCHS
Gm12G015300
Gm20G169200
Gm11G070500
Gm07G243651
R
N
Phloem
Phloem fiber
Vascular
Cambium
Endodermis
Procambium
Xylem
Pericycle
Vascular bundle
Cortex
Epidermis
Average expression
0
1
B
Cortex
Gm07G178200
Gm03G029100
GmCYP715A1
GmUSPL1
GmWRKY6.4
Gm07G039700
GmFQR1
GmCWINV1
Gm02G298100
Gm03G248400
R
N
Phloem
Phloem fiber
Vascular
Cambium
Endodermis
Procambium
Xylem
Pericycle
Vascular bundle
Cortex
Epidermis
Average expression
0
1

Figure S5

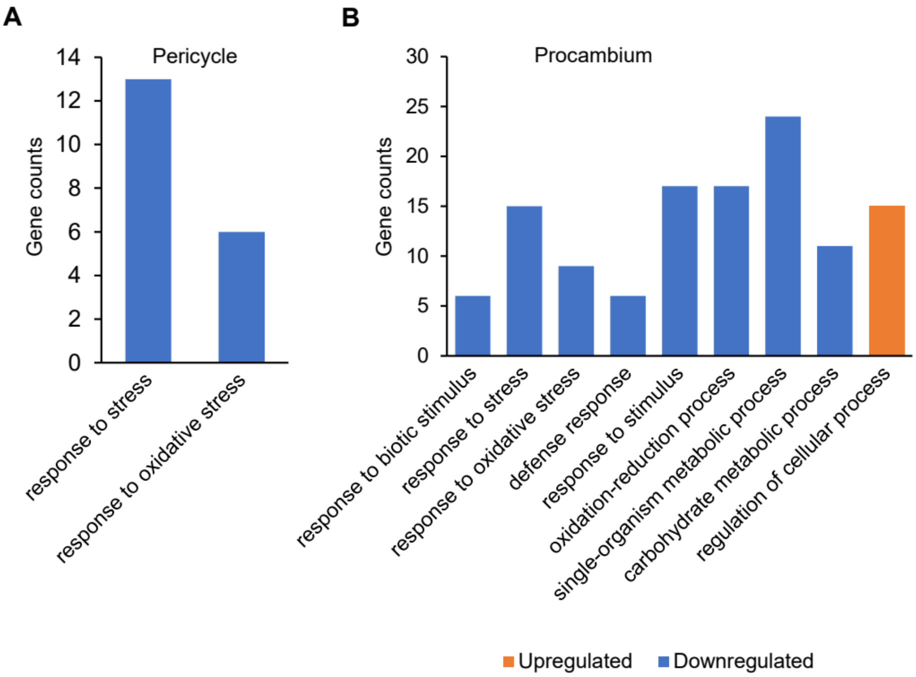
A
Pericycle
Gene counts
14
12
10
8
6
4
2
0
response to stress
response to oxidative stress
B
Procambium
Gene counts
30
25
20
15
10
5
0
response to biotic stimulus
response to stress
response to oxidative stress
defense response
response to stimulus
oxidation-reduction process
single-organism metabolic process
carbohydrate metabolic process
regulation of cellular process
Upregulated
Downregulated

Figure S6

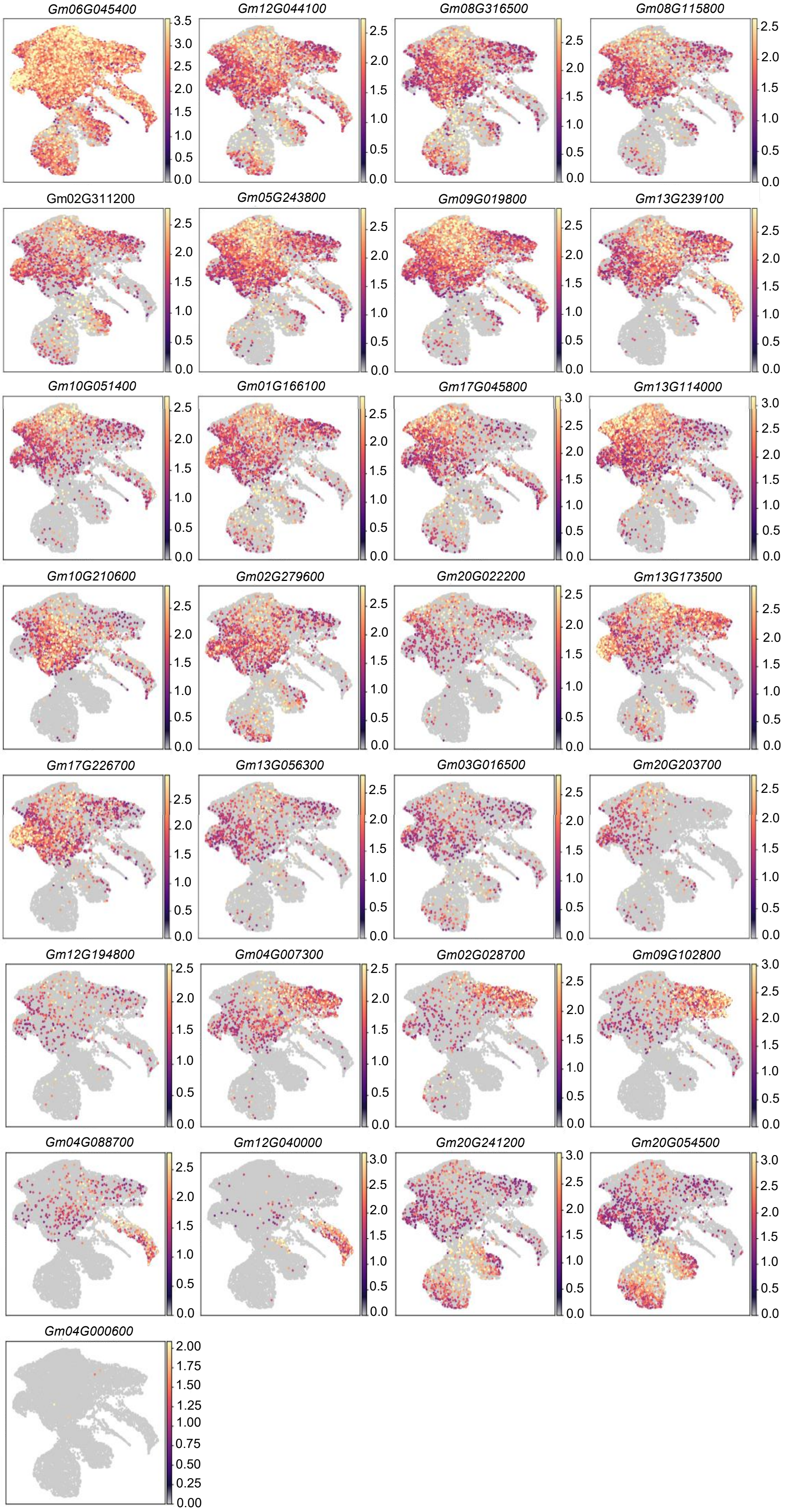
Gm06G045400
Gm12G044100
Gm08G316500
Gm08G115800
Gm02G311200
Gm05G243800
Gm09G019800
Gm13G239100
Gm10G051400
Gm01G166100
Gm17G045800
Gm13G114000
Gm10G210600
Gm02G279600
Gm20G022200
Gm13G173500
Gm17G226700
Gm13G056300
Gm03G016500
Gm20G203700
Gm12G194800
Gm04G007300
Gm02G028700
Gm09G102800
Gm04G088700
Gm12G040000
Gm20G241200
Gm20G054500
Gm04G000600

Figure S7

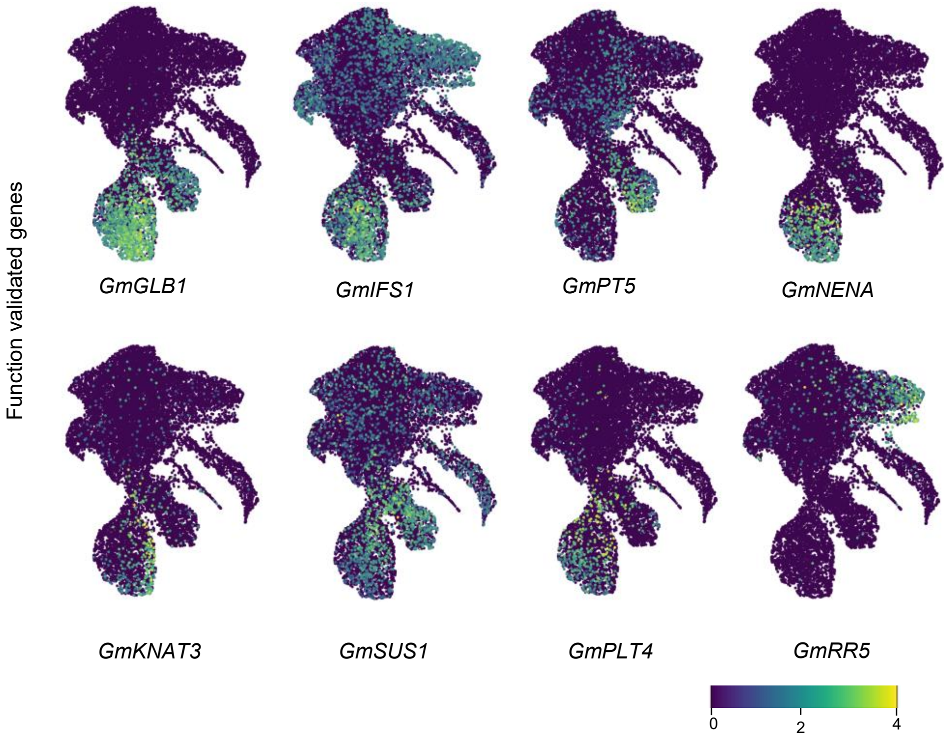
Function validated genes
GmGLB1
GmIFS1
GmPT5
GmNENA
GmKNAT3
GmSUS1
GmPLT4
GmRR5
0
2
4

Figure S8

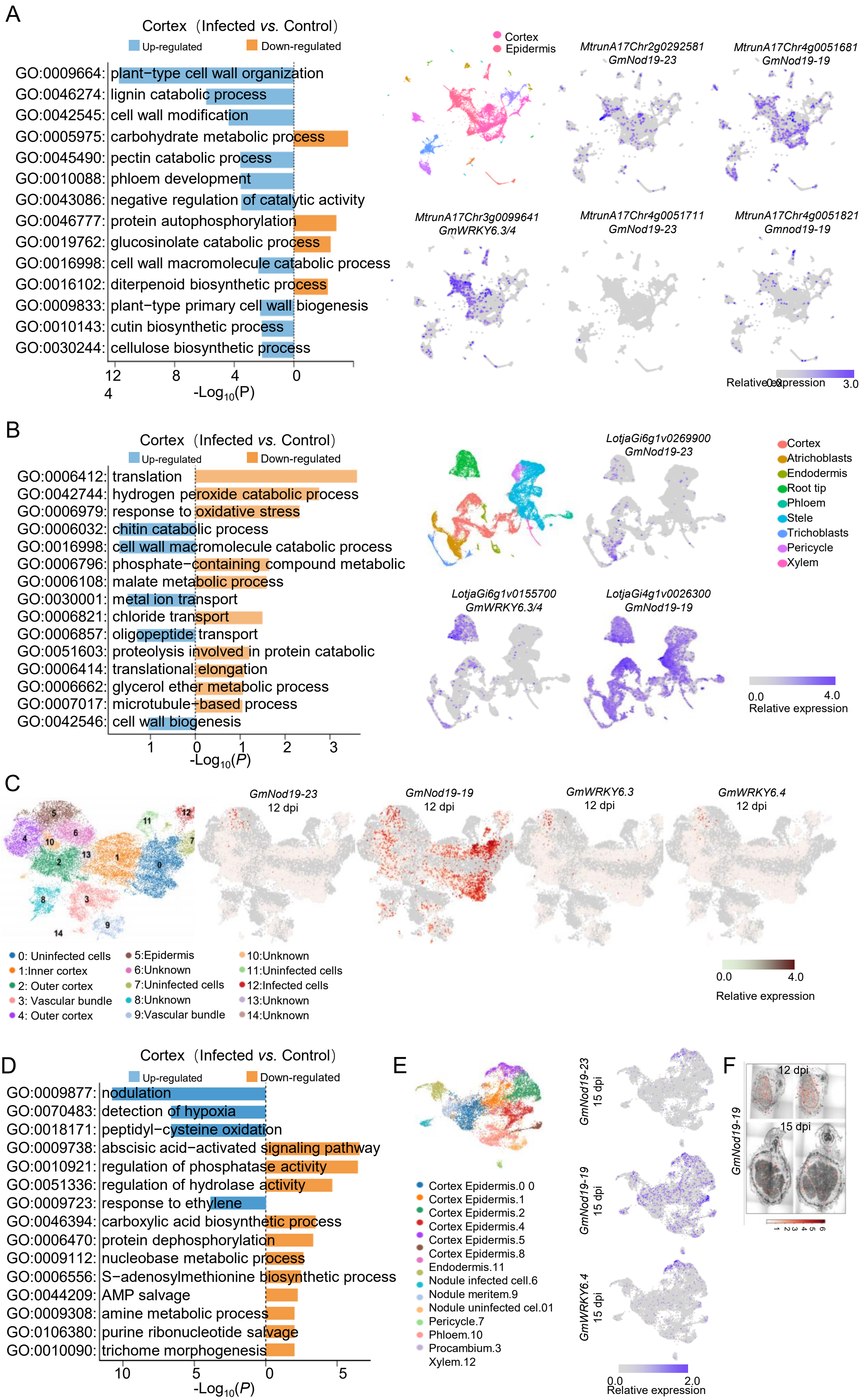
A
Cortex (Infected vs. Control)
Up-regulated
Down-regulated
GO:0009664: plant−type cell wall organization
GO:0046274: lignin catabolic process
GO:0042545: cell wall modification
GO:0005975: carbohydrate metabolic process
GO:0045490: pectin catabolic process
GO:0010088: phloem development
GO:0043086: negative regulation of catalytic activity
GO:0046777: protein autophosphorylation
GO:0019762: glucosinolate catabolic process
GO:0016998: cell wall macromolecule catabolic process
GO:0016102: diterpenoid biosynthetic process
GO:0009833: plant−type primary cell wall biogenesis
GO:0010143: cutin biosynthetic process
GO:0030244: cellulose biosynthetic process
12
8
4
0
4
-Log10(P)
Cortex
Epidermis
MtrunA17Chr2g0292581
GmNod19-23
MtrunA17Chr4g0051681
GmNod19-19
MtrunA17Chr3g0099641
GmWRKY6.3/4
MtrunA17Chr4g0051711
GmNod19-23
MtrunA17Chr4g0051821
Gmnod19-19
Relative expression
0.0
3.0
B
Cortex (Infected vs. Control)
Up-regulated
Down-regulated
GO:0006412: translation
GO:0042744: hydrogen peroxide catabolic process
GO:0006979: response to oxidative stress
GO:0006032: chitin catabolic process
GO:0016998: cell wall macromolecule catabolic process
GO:0006796: phosphate−containing compound metabolic
GO:0006108: malate metabolic process
GO:0030001: metal ion transport
GO:0006821: chloride transport
GO:0006857: oligopeptide transport
GO:0051603: proteolysis involved in protein catabolic
GO:0006414: translational elongation
GO:0006662: glycerol ether metabolic process
GO:0007017: microtubule−based process
GO:0042546: cell wall biogenesis
1
0
1
2
3
-Log10(P)
LotjaGi6g1v0269900
GmNod19-23
Cortex
Atrichoblasts
Endodermis
Root tip
Phloem
Stele
Trichoblasts
Pericycle
Xylem
LotjaGi6g1v0155700
GmWRKY6.3/4
LotjaGi4g1v0026300
GmNod19-19
0.0
4.0
Relative expression
C
GmNod19-23
12 dpi
GmNod19-19
12 dpi
GmWRKY6.3
12 dpi
GmWRKY6.4
12 dpi
0: Uninfected cells
1:Inner cortex
2: Outer cortex
3: Vascular bundle
4: Outer cortex
5:Epidermis
6:Unknown
7:Uninfected cells
8:Unknown
9:Vascular bundle
10:Unknown
11:Uninfected cells
12:Infected cells
13:Unknown
14:Unknown
0.0
4.0
Relative expression
D
Cortex (Infected vs. Control)
Up-regulated
Down-regulated
GO:0009877: nodulation
GO:0070483: detection of hypoxia
GO:0018171: peptidyl−cysteine oxidation
GO:0009738: abscisic acid−activated signaling pathway
GO:0010921: regulation of phosphatase activity
GO:0051336: regulation of hydrolase activity
GO:0009723: response to ethylene
GO:0046394: carboxylic acid biosynthetic process
GO:0006470: protein dephosphorylation
GO:0009112: nucleobase metabolic process
GO:0006556: S−adenosylmethionine biosynthetic process
GO:0044209: AMP salvage
GO:0009308: amine metabolic process
GO:0106380: purine ribonucleotide salvage
GO:0010090: trichome morphogenesis
10
5
0
5
-Log10(P)
E
Cortex Epidermis.0 0
Cortex Epidermis.1
Cortex Epidermis.2
Cortex Epidermis.4
Cortex Epidermis.5
Cortex Epidermis.8
Endodermis.11
Nodule infected cell.6
Nodule meritem.9
Nodule uninfected cel.01
Pericycle.7
Phloem.10
Procambium.3
Xylem.12
GmNod19-23
15 dpi
GmNod19-19
15 dpi
GmWRKY6.4
15 dpi
0.0
2.0
Relative expression
F
12 dpi
15 dpi
GmNod19-19
1
2
3
4
5
6

Figure S9

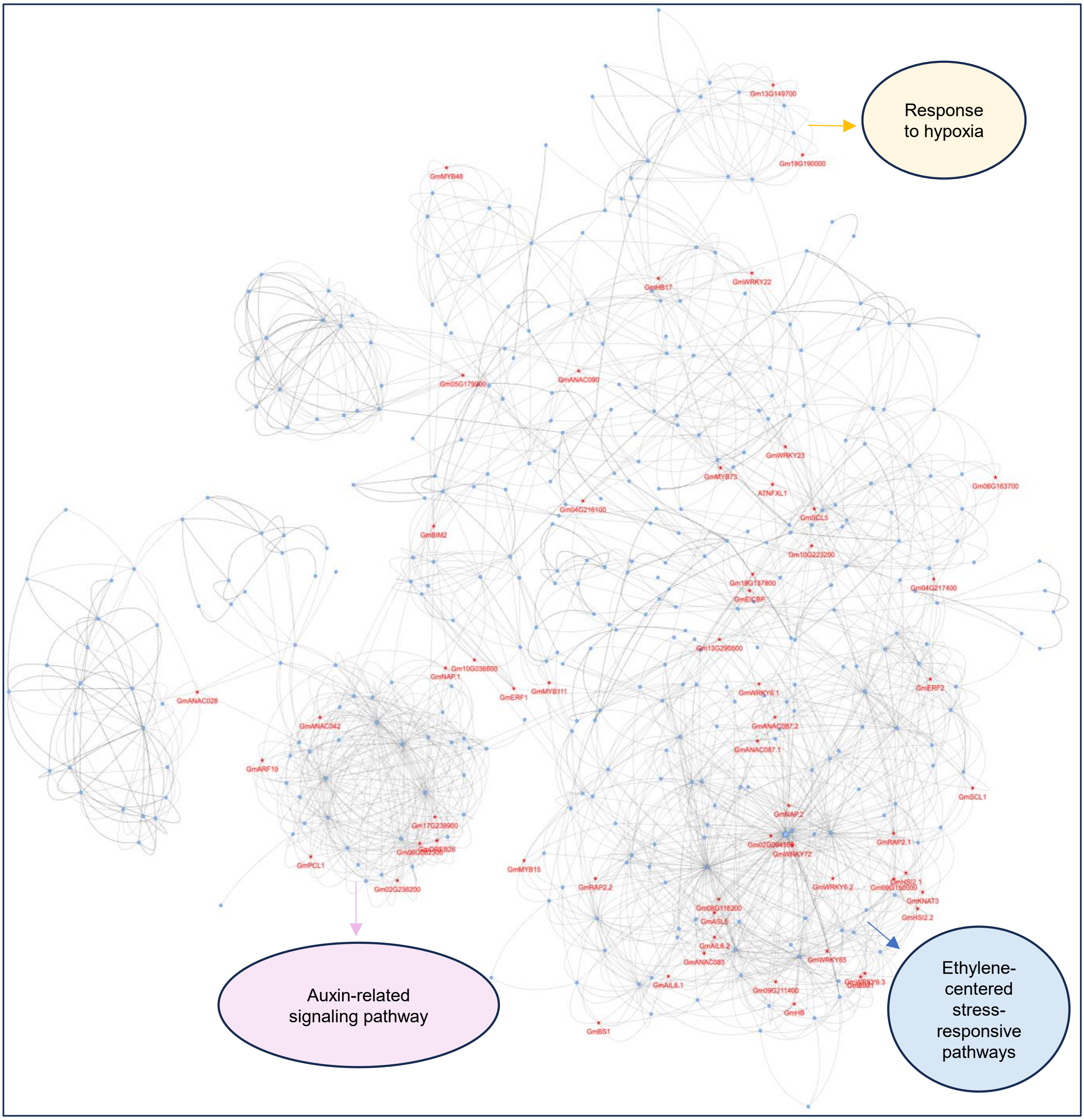
Response
to hypoxia
Auxin-related
signaling pathway
Ethylene-
centered
stress-
responsive
pathways
Gm13G149700
Gm19G190000
GmMYB48
GmHB17
GmWRKY22
Gm05G179900
GmANAC090
GmWRKY23
GmMYB73
ATNFXL1
Gm06G163700
Gm04G216100
GmSCL5
GmBIM2
Gm10G223200
Gm19G137800
Gm04G217400
Gm13G290500
Gm10G036600
GmNAP.1
GmERF1
GmMYB111
GmANAC028
GmWRKY6.1
GmERF2
GmANAC042
GmANAC087.2
GmANAC087.1
GmARF19
GmSCL1
GmNAP.2
Gm17G239900
GmWRKY72
GmRAP2.1
GmPCL1
GmMYB15
Gm02G236200
GmRAP2.2
GmWRKY6.2
GmKNAT3
GmHSI2.2
Gm08G118200
GmASL5
GmAIL6.2
GmANAC083
GmWRKY65
GmAIL6.1
Gm09G211400
GmHB
GmBS1

Figure S10

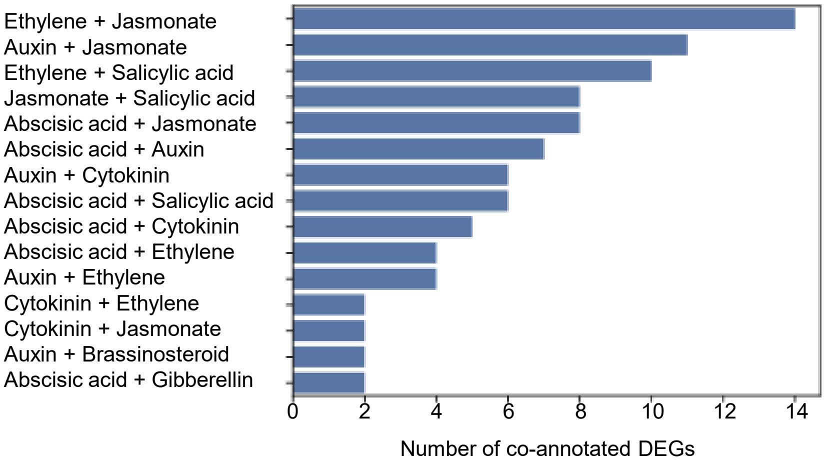
Ethylene + Jasmonate
Auxin + Jasmonate
Ethylene + Salicylic acid
Jasmonate + Salicylic acid
Abscisic acid + Jasmonate
Abscisic acid + Auxin
Auxin + Cytokinin
Abscisic acid + Salicylic acid
Abscisic acid + Cytokinin
Abscisic acid + Ethylene
Auxin + Ethylene
Cytokinin + Ethylene
Cytokinin + Jasmonate
Auxin + Brassinosteroid
Abscisic acid + Gibberellin
0
2
4
6
8
10
12
14
Number of co-annotated DEGs

Figure S11

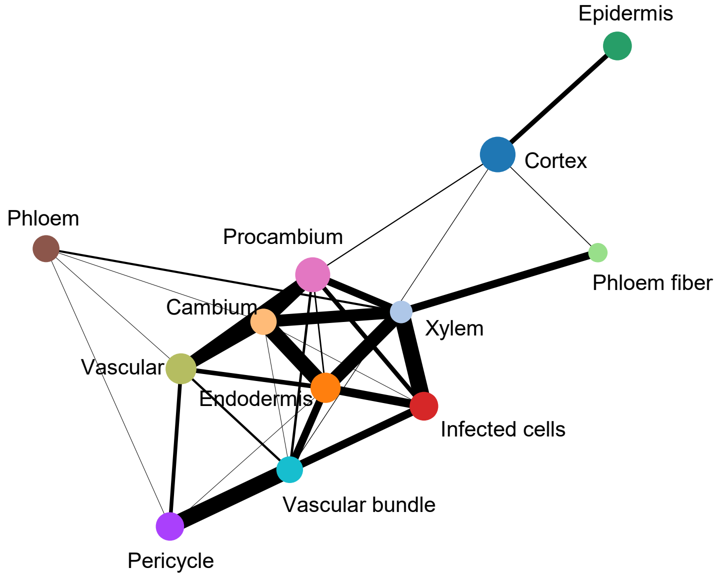
Epidermis
Cortex
Phloem
Procambium
Phloem fiber
Cambium
Xylem
Vascular
Endodermis
Infected cells
Vascular bundle
Pericycle

Figure S12

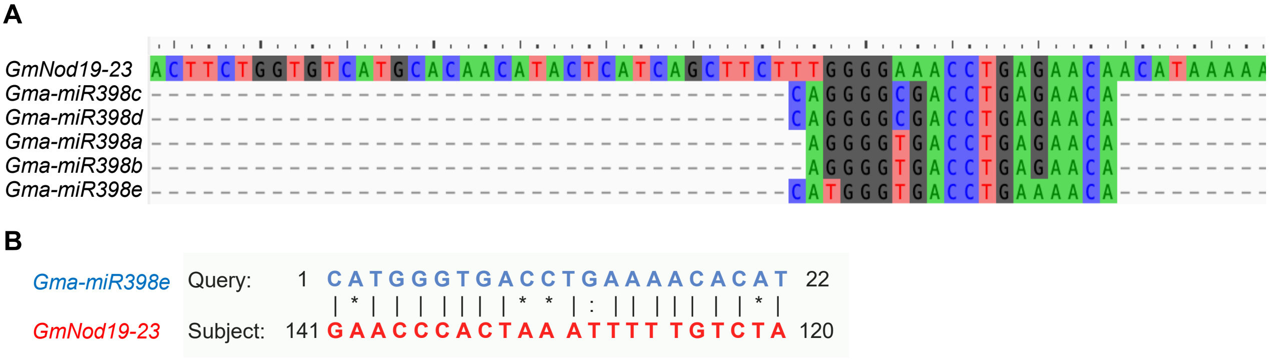
A
GmNod19-23 ACTTCTGGTGTCATGCACAACATACTCATCAGCTTCTTTGGGGAAACCTGAGAACAACATAAAAA
Gma-miR398c CAGGGGCGACCTGAGAACA
Gma-miR398d CAGGGGCGACCTGAGAACA
Gma-miR398a AGGGGTGACCTGAGAACA
Gma-miR398b AGGGGTGACCTGAGAACA
Gma-miR398e CATGGGTGACCTGAAAACA
B
Gma-miR398e Query: 1 CATGGGTGACCTGAAAACACAT 22
| * | | | | | | | * * | : | | | | | | | * |
GmNod19-23 Subject: 141 GAACCCACTAA ATTTT TGTCTA 120

Figure S13

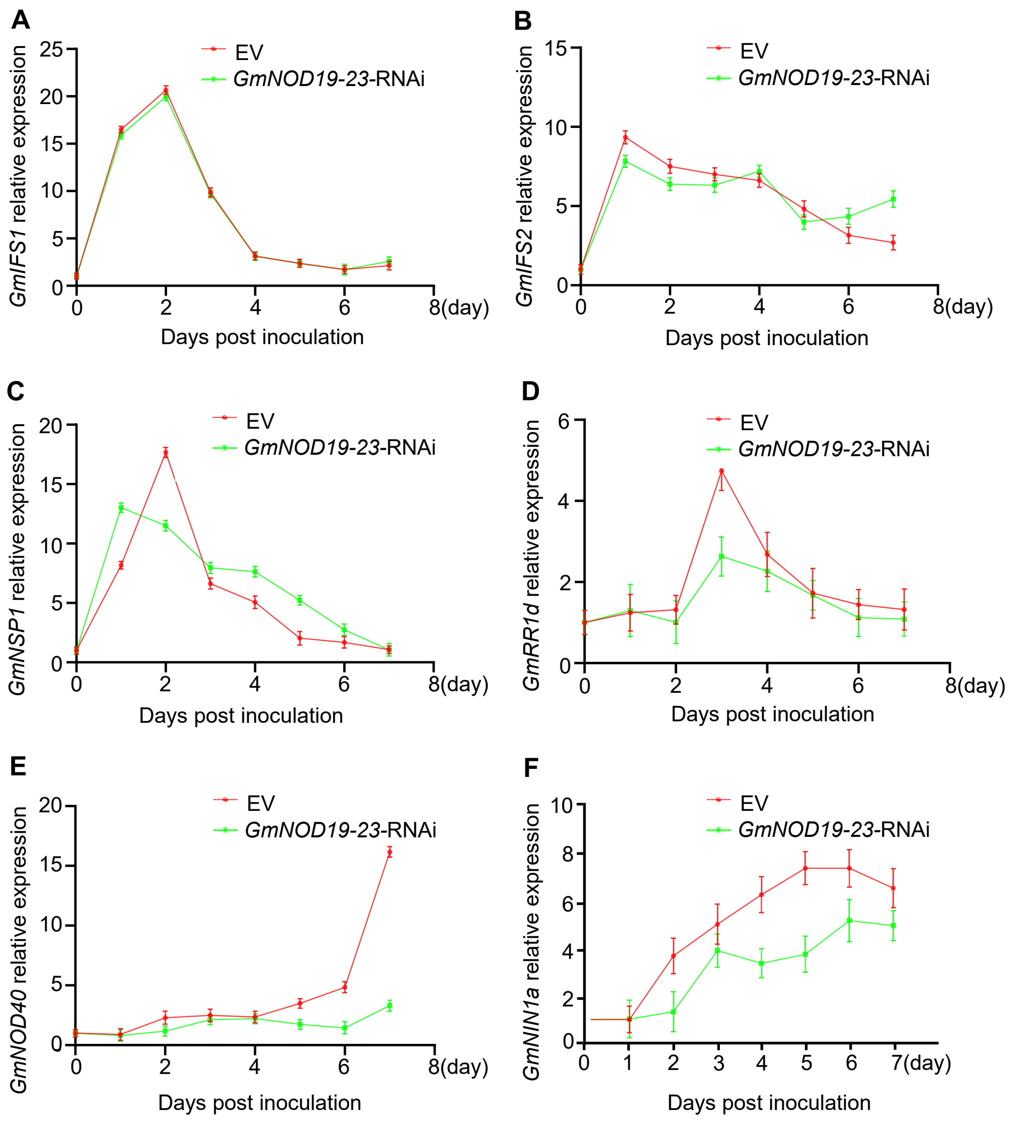

A
B
C
D
E
F
EV
GmNOD19-23-RNAi
GmIFS1 relative expression
GmIFS2 relative expression
GmNSP1 relative expression
GmRR1d relative expression
GmNOD40 relative expression
GmNIN1a relative expression
Days post inoculation
8(day)
7(day)

Figure S14

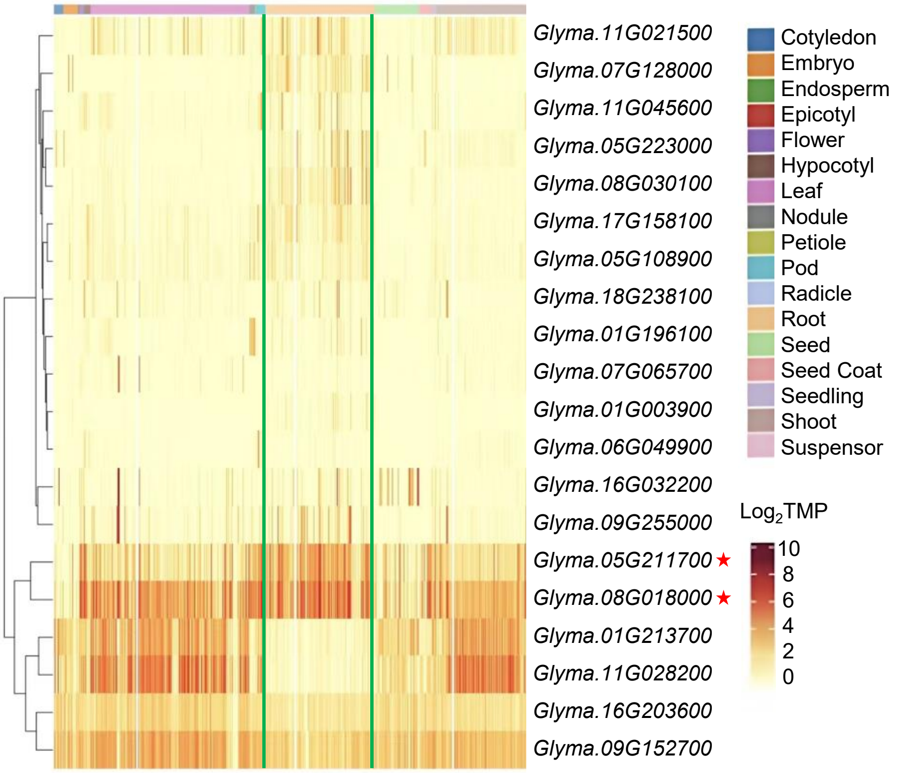
Glyma.11G021500
Glyma.07G128000
Glyma.11G045600
Glyma.05G223000
Glyma.08G030100
Glyma.17G158100
Glyma.05G108900
Glyma.18G238100
Glyma.01G196100
Glyma.07G065700
Glyma.01G003900
Glyma.06G049900
Glyma.16G032200
Glyma.09G255000
Glyma.05G211700 ★
Glyma.08G018000 ★
Glyma.01G213700
Glyma.11G028200
Glyma.16G203600
Glyma.09G152700
Cotyledon
Embryo
Endosperm
Epicotyl
Flower
Hypocotyl
Leaf
Nodule
Petiole
Pod
Radicle
Root
Seed
Seed Coat
Seedling
Shoot
Suspensor
Log2TMP
10
8
6
4
2
0

Figure S15

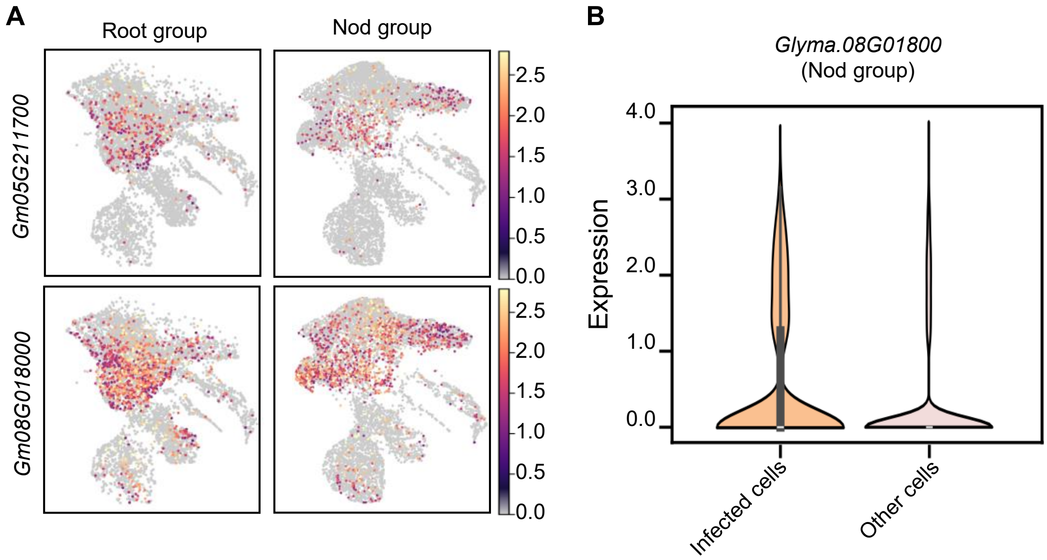

A
Root group
Nod group
Gm05G211700
Gm08G018000
2.5
2.0
1.5
1.0
0.5
0.0
B
Glyma.08G01800
(Nod group)
Expression
4.0
3.0
2.0
1.0
0.0
Infected cells
Other cells

Figure S16

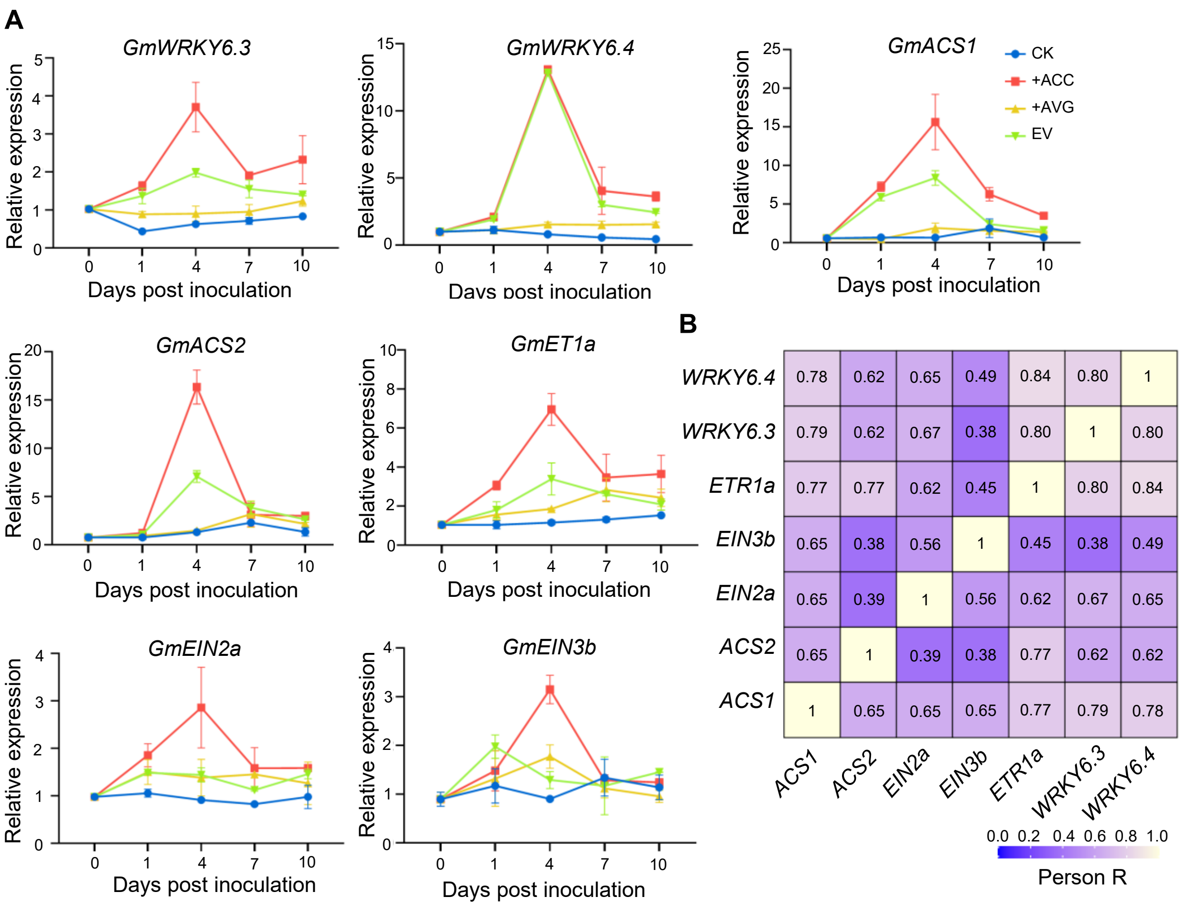
A
GmWRKY6.3
GmWRKY6.4
GmACS1
GmACS2
GmET1a
GmEIN2a
GmEIN3b
Relative expression
Days post inoculation
CK
+ACC
+AVG
EV
B
WRKY6.4 0.78 0.62 0.65 0.49 0.84 0.80 1
WRKY6.3 0.79 0.62 0.67 0.38 0.80 1 0.80
ETR1a 0.77 0.77 0.62 0.45 1 0.80 0.84
EIN3b 0.65 0.38 0.56 1 0.45 0.38 0.49
EIN2a 0.65 0.39 1 0.56 0.62 0.67 0.65
ACS2 0.65 1 0.39 0.38 0.77 0.62 0.62
ACS1 1 0.65 0.65 0.65 0.77 0.79 0.78
ACS1 ACS2 EIN2a EIN3b ETR1a WRKY6.3 WRKY6.4
0.0 0.2 0.4 0.6 0.8 1.0
Person R

Figure S17

A

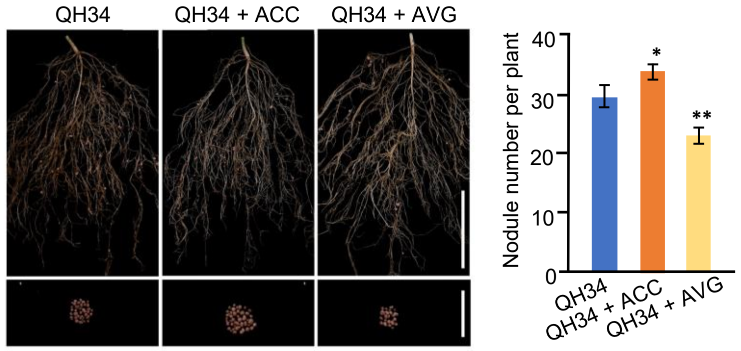

B

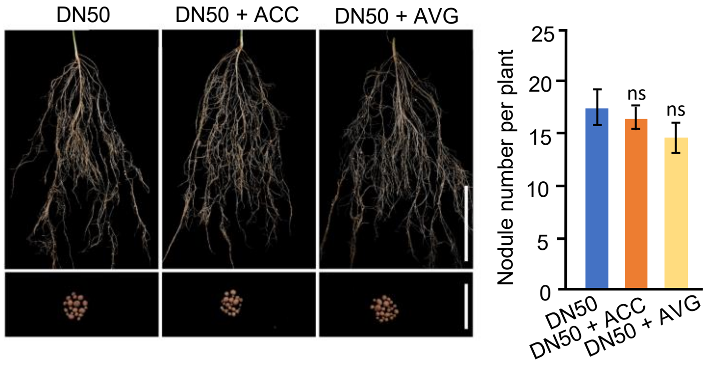

Figure S18

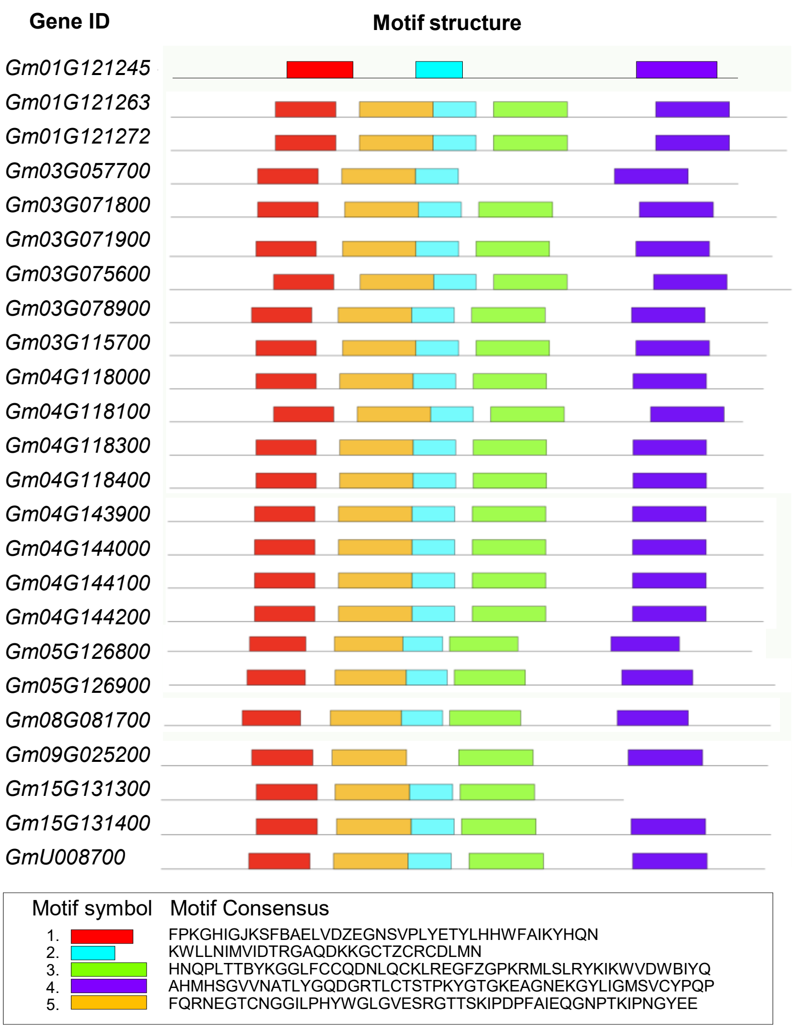
Gene ID
Motif structure
Gm01G121245
Gm01G121263
Gm01G121272
Gm03G057700
Gm03G071800
Gm03G071900
Gm03G075600
Gm03G078900
Gm03G115700
Gm04G118000
Gm04G118100
Gm04G118300
Gm04G118400
Gm04G143900
Gm04G144000
Gm04G144100
Gm04G144200
Gm05G126800
Gm05G126900
Gm08G081700
Gm09G025200
Gm15G131300
Gm15G131400
GmU008700
Motif symbol
Motif Consensus
1. FPKGHIGJKSFBAELVDZEGNSVPLYETYLHHWFAIKYHQN
2. KWLLNIMVIDTRGAQDKKGCTZCRCDLMN
3. HNQPLTTBYKGGLFCCQDNLQCKLREGFZGPKRMLSLRYKIKWVDWBIYQ
4. AHMHSGVVNATLYGQDGRTLCTSTPKYGTGKEAGNEKGYLIGMSVCYPQP
5. FQRNEGTCNGGILPHYWGLGVESRGTTSKIPDPFAIEQGNPTKIPNGYEE

Figure S19

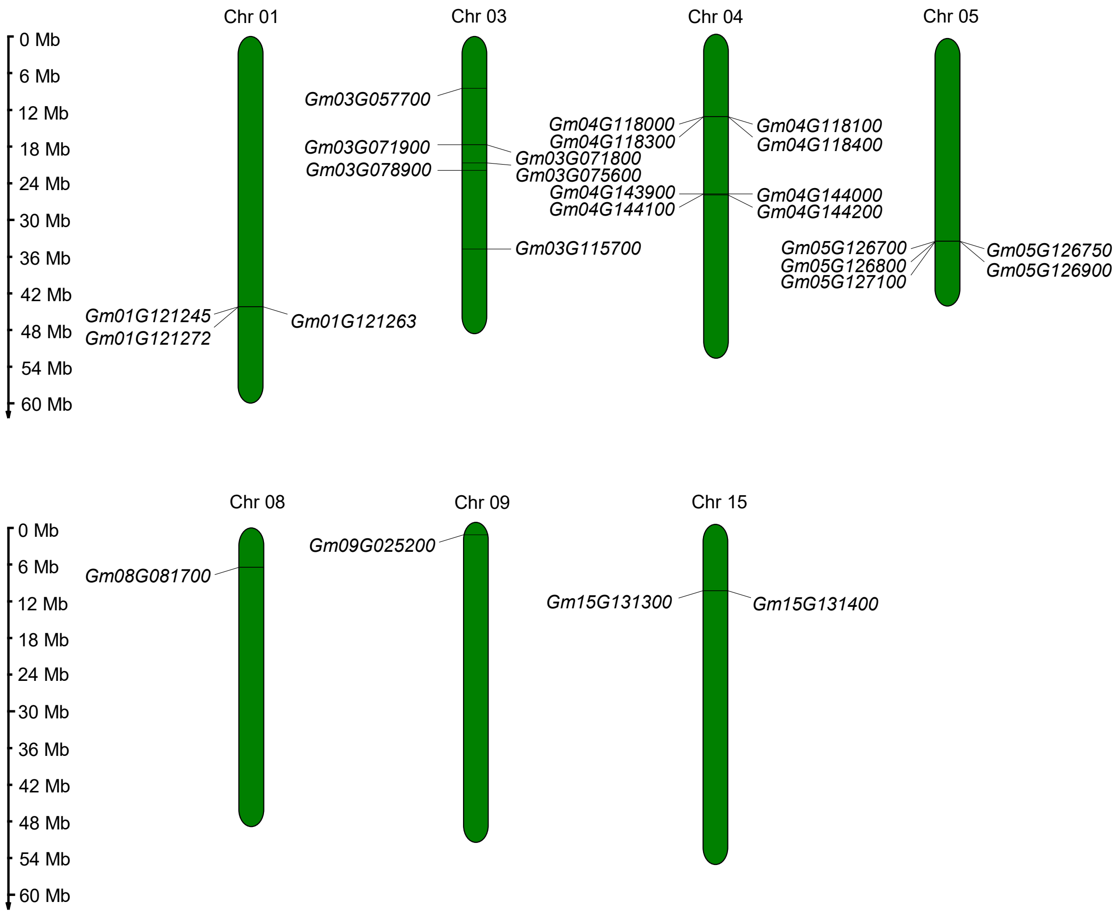
Chr 01
Chr 03
Chr 04
Chr 05
0 Mb
6 Mb
12 Mb
18 Mb
24 Mb
30 Mb
36 Mb
42 Mb
48 Mb
54 Mb
60 Mb
Gm01G121245
Gm01G121272
Gm01G121263
Gm03G057700
Gm03G071900
Gm03G078900
Gm03G071800
Gm03G075600
Gm03G115700
Gm04G118000
Gm04G118300
Gm04G118100
Gm04G118400
Gm04G143900
Gm04G144100
Gm04G144000
Gm04G144200
Gm05G126700
Gm05G126800
Gm05G127100
Gm05G126750
Gm05G126900
Chr 08
Chr 09
Chr 15
0 Mb
6 Mb
12 Mb
18 Mb
24 Mb
30 Mb
36 Mb
42 Mb
48 Mb
54 Mb
60 Mb
Gm08G081700
Gm09G025200
Gm15G131300
Gm15G131400

Figure S20

```
Gm05G126700  ------------------------------------------------------------
Gm05G126750  MRFLKVLPLFESIVCSCYIIQSRLKVYFSALNRVCGSSPVLDFFIAEDYKRDQHGFCISR
Gm05G127100  ------------------------------------------MF-----------F----
Gm05G126900  ----------------------------------------MDFF-----------FPLAI

Gm05G126700  ------------------------------------------------------------
Gm05G126750  LVIFIGNTCGYFEDFGKTESNMKVKSTVFLSPKFELGPGSATTRHYYDIEFPRGHVALKS
Gm05G127100  ------------------------------------------------------------
Gm05G126900  LVMILGTP--YSSASGKSESN--VKTAVFLSPKFELGPGSVANKYDYDIDFPRGHIALKS

Gm05G126700  ------------------------------------------------------------
Gm05G126750  FSGEVVDEAGNPVPLHETYLHHWIV-----------------------------------
Gm05G127100  ------------------------------------------------------------
Gm05G126900  FNAEVVDEAGNPVPLHETYLHHWVVGRYYQPKYVTTHTNYDGHRILHNSDHIFVRNSGIC

Gm05G126700  --------------------------------------------MIKVHAIDTRGLVDKM
Gm05G126750  ------------------------------------------------------------
Gm05G127100  ------------------------------------------------------------
Gm05G126900  QRDILGQYYGLGSETRGTATDVPDPFGIVVGDHAEIPEGYEEKWLVNIHAIDTRGVVDKM

Gm05G126700  GCTECKCDLYNVTKDENGEPLRPDYKGGMFCCYDQMQCRLREGFDGTKRSLYLRYT----
Gm05G126750  ----------------------------------------------------VRYT----
Gm05G127100  ------------------------------------------------------------
Gm05G126900  GCTECRCDLYNVTKDEYGEFLRPDYKGGLKCCYDQTQCRLREGFEGPKRSLYLRYTVKWV

Gm05G126700  ----------------------------------------VESCSTDHKDGNGCLDVKRT
Gm05G126750  ----------------------------------------IN------------------
Gm05G127100  ------------------------------------------------------------
Gm05G126900  EWDKFIVPVKIYILDVTDTLKISDDSGEMIPEHDCRVEYEVEYCSTGQKNGNGCLDGKRT

Gm05G126700  SLPMKKGGYVIYGVAHQRSGWKGYMLF---------------------------------
Gm05G126750  ------------------------------------------------------------
Gm05G127100  ---------------------------------------------------------NRC
Gm05G126900  SLPIQKGGYVIYGVAHQHSGGTGSTLYGQDGRVICSSIPSYGKGKEAGNEADYIVGMSTC

Gm05G126700  -----NTKI---------WKWKRSRK----------------------------------
Gm05G126750  -----NVKM---------------------------------------------------
Gm05G127100  YPQPGSVKIIDGETLTLESNYSSSREHTGVMGLFYLLVAEQLPHQHFRHTPRSSFSMDIN
Gm05G126900  YPRPGSVKIIDGETLTLESNYSSSREHTGVMGLFYLLVAEQLPDQHFRHTSRSSFFMNIN
                  ..*:

Gm05G126700  ----
Gm05G126750  ----
Gm05G127100  SIFH
Gm05G126900  SIFH
```

Figure S21

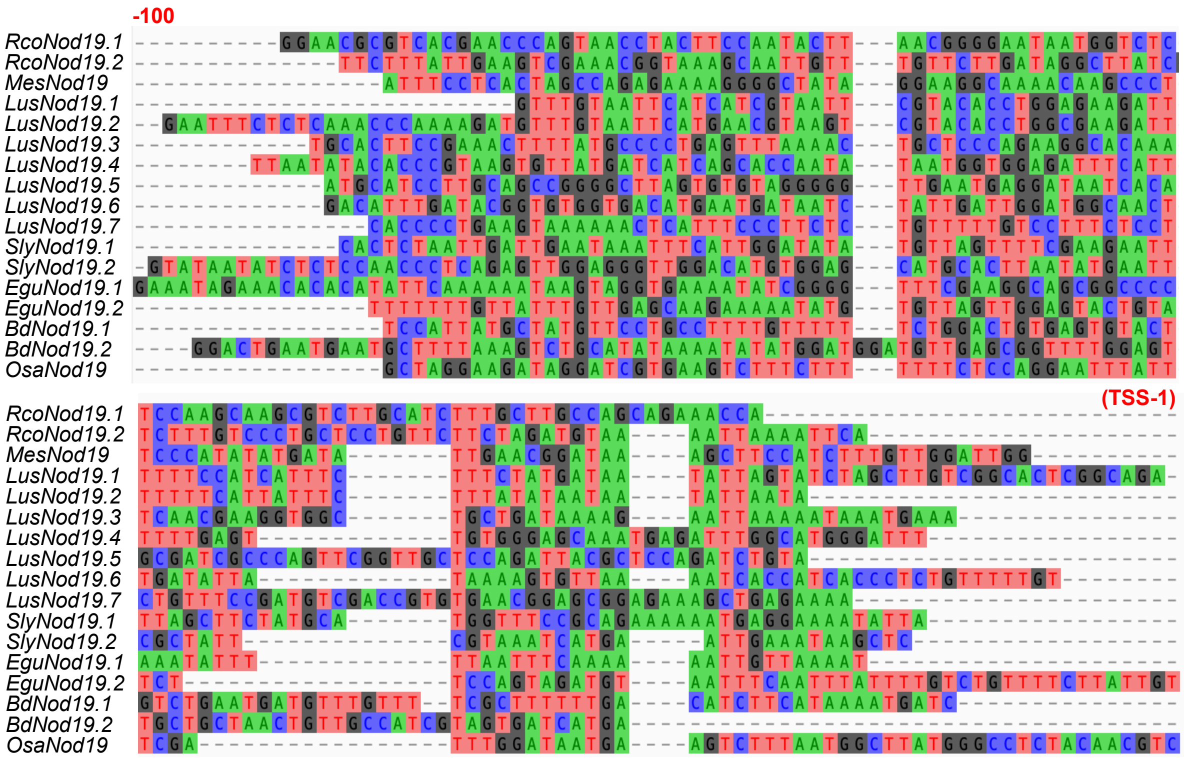
-100
(TSS-1)
RcoNod19.1
RcoNod19.2
MesNod19
LusNod19.1
LusNod19.2
LusNod19.3
LusNod19.4
LusNod19.5
LusNod19.6
LusNod19.7
SlyNod19.1
SlyNod19.2
EguNod19.1
EguNod19.2
BdNod19.1
BdNod19.2
OsaNod19

Figure S22
