# Supplementary material for: Single‐cell Transcriptome Profiling Reveals Gene Regulatory Networks and Key Genes in the Root Epidermis and Cortical Cells Associated with Early Nodulation in Glycine Max
Source: Adv Sci (Weinh). 2026 Jul 11:e76550. Online ahead of print. doi: 10.1002/advs.76550 (PMC13355928; doi:10.1002/advs.76550)
Supplement: Supplementary file 3 — Supporting File 3: advs76550‐sup‐0003‐Figure and tables captions.docx. [file ADVS-9999-e76550-s003.docx]

**Supporting Information**

**Supplemental figures**

**Figure S1. UMAP visualization of the expression patterns of cell type marker genes in R and N groups.** Each dot represents an individual cell. Color intensity indicates the normalized expression level of each marker gene in individual cells.

**Figure S2. Cell identity classification of infected cells. (A)** UMAP clustering of R group cells. **(B)** UMAP clustering of N group cells. **(C)** Donut plot showing the agreement between Scanpy and Decoupler cell identity assignments. **(D)** Correlation analysis of cell identity assignments between Scanpy and Decoupler. Black triangles indicate previously identified infected cells.

**Figure S3. Proportion of differentially expressed genes shared by different cell types between the R and N groups**.

**Figure S4**. **Differential expression analysis of genes shared between cortex and epidermis.**

**(A)** Response patterns of shared DEGs between inoculated and control conditions in cortex and epidermis. **(B)** GO enrichment analysis (Biological Process) of shared DEGs. Bubble size indicates gene count, color indicates *P value* (Fisher's exact test), and the x-axis shows gene ratio (%).

**Figure S5. Heatmap representation of top-10 most significant DEGs in epidemical and cortical cells.** DEGs were ranked based on the p-value between root cells inoculated with B. japonicum (N group) or mock-inoculated (R group) in the epidermis **(A)** and cortex **(B)**.

**Figure S6. Enriched gene-ontology terms in pericycle and procambium cells. (A, B)** Enriched gene-ontology (GO) terms in pericycle cells **(A)** and procambium cells (**B**). The number of up- or down-regulated genes enriched in the indicated pathways is shown in different colors.

**Figure S7. UMAP plot showing 29 function-validated SNF genes showed no differential expression in current study**. Each dot represents an individual cell. Color intensity indicates the normalized expression level of each gene in individual cells.

**Figure S8. UMAP plot showing 8 function-validated SNF genes identified as DEGs in current study.** Each dot represents an individual cell. Color intensity indicates the normalized expression level of each gene in individual cells.

**Figure S9. Cross-species comparison of single-cell datasets from rhizobial infection experiments in soybean, *Medicago truncatula*, and *Lotus japonicus*. (A)** GO biological process (GO_BP) enrichment of cortical DEGs between inoculated and control groups at 4 days post-inoculation in M. truncatula, together with expression profiles of homologs of GmWRKY6.3, GmWRKY6.4, Nod19-23, and Nod19-19. **(B)** GO_BP enrichment of cortical DEGs between inoculated and control groups at 5 days post-inoculation in L. japonicus, together with expression profiles of homologs of GmWRKY6.3, GmWRKY6.4, Nod19-23, and Nod19-19. **(C, D)** GO_BP enrichment of cortical DEGs between inoculated and control groups at 12 days post-inoculation in soybean, together with expression profiles of GmWRKY6.3, GmWRKY6.4, Nod19-23, and Nod19-19. **(E)** Expression profiles of GmWRKY6.3, GmWRKY6.4, Nod19-23, and Nod19-19 in soybean root at 15 days post-inoculation.

**Figure S10. Gene regulatory network inferred for 58 transcription-factor genes differentially expressed in root cells inoculated with *B. japonicum* (R group) or mock-inoculated (N group).** Red stars indicate each transcription-factor gene and blue circles indicate genes involved in their regulatory networks. Three clusters associated with known signaling pathways are shown in colored ellipses.

**Figure S11. Hormones crosstalk analysis based on differentially expressed genes with co-annotated GO terms involved in different hormone pathways.**

**Figure S12. PAGA analysis to determine connectivity, based on a threshold of 0.03 for the annotated cell types.**

**Figure S13. Multiple sequence alignment and target prediction of *Gma-miR398* and *GmNod19-23*. (A)** Multiple sequence alignment of *GmNod19-23* and predicted miR398s in soybean. **(B)** Target prediction of *GmNod19-23* by *miR298e*.

**Figure S14. Expression dynamics of infection-stage molecular markers. (A–C)** Markers for pre-infection signaling (*GmIFS1*, *GmIFS2, GmNSP1*) and nodule-primordium development (**D–F**) (*GmRR1d*, *GmENOD40*, *GmNINa*) were assayed by qRT–PCR and compared between EV and *GmNod19-23* RNAi lines at 1-7 dpi.

**Figure S15. Tissue-specific expression of soybean ACC synthase (*ACS*) genes.** Heatmap showing the expression profiles of 20 annotated soybean *ACS* genes across different tissues, based on data obtained from SoyBase.

**Figure S16. Expression pattern and quantitative analysis of the key rate-limiting ethylene biosynthesis enzyme GmACS2 in inoculated and control groups. (A)** UMAP showing GmACS2 expression in the R and N groups. **(B)** Student’s t-test (*P* < 0.05) comparing ACS2 expression levels between infected cells and other cell types.

**Figure S17. Expression dynamics of key ethylene signaling pathway genes after exogenous application of the ethylene precursor ACC and the ethylene biosynthesis inhibitor AVG. (A)** Time-course line plots of ethylene signaling pathway gene expression. **(B)** Correlation heatmap of ethylene signaling pathway gene expression. CK, the untreated control group. EV, the rhizobium-inoculated group without ACC/AVG treatment.

**Figure S18. Effects of exogenous ACC (ethylene precursor) and AVG (ethylene biosynthesis inhibitor) on nodulation in different soybean cultivars. (A, B)** Nodulation phenotype **(A)** and nodule number per plant **(B)** of Qi Huang 34. **(C, D)** Nodulation phenotype **(C)** and nodule number per plant **(D)** of DongNong 50. Scale bars = 8 cm. Data are presented as mean ± SD. Statistical significance was determined by two-tailed Student's t-test (*P <* 0.05).

**Figure S19. MEME motif search of the 24 annotated GmNod19 proteins.**

**Figure S20. Chromosomal distribution of *GmNod19* genes identified in soybean.**

**Figure S21. Multiple sequence alignment of *Gm05G126700*, *Gm05G126750*, *Gm05G127100*, and *Gm05G126900*.**

**Figure S22. Multiple sequence alignment of the promoter regions of *Nod19* genes in representative species.**

**Supplemental tables**

**Table S1** Quality assessment of scRNA-seq experiments.

**Table S2** Markers used for identification of cell types.

**Table S3** Top marker genes identified in each cell cluster.

**Table S4** Cell types-specific markers that were significant in both R and N conditions.

**Table S5** The proportions of cells from different treatments in each cluster.

**Table S6** Expression profiling and function annotation of identified cell-type-specific DEGs responsive to *B. japonicum* infection.

**Table S7** GO enrichment analysis of DEGs in epidermal, cortical and infected cells.

**Table S8** DEGs identified for infected cells related comparision in N group dataset.

**Table S9** Transcription factors and their associated genes in the constructed GRNs.

**Table S10** Primers used in current study.

**Table S11** Species used in comparative analysis of stress up-regulated *Nod19* gene family evolution.

**Table S12** Identified genes belong to the stress up-regulated *Nod19* family in each species.
